# Aryl Viologens: Unprecedented Stability of Viologen-Derivatives as Anolytes for Alkaline Redox Flow Batteries

**DOI:** 10.1021/acsaem.5c02255

**Published:** 2025-11-03

**Authors:** Rubén Rubio-Presa, Edgar Ventosa, Roberto Sanz

**Affiliations:** † Department of Chemistry, 16725 University of Burgos, Plaza Misael Bañuelos s/n, Burgos E-09001, Spain; ‡ International Research Centre in Critical Raw Materials-ICCRAM, University of Burgos, Plaza Misael Bañuelos s/n, Burgos E-09001, Spain

**Keywords:** aqueous organic redox flow batteries, alkaline media, aryl viologen, chemical stability, molecular engineering

## Abstract

Viologen derivatives are widely used in the anolytes of aqueous organic redox flow batteries (AORFBs). However, their applications have been restricted to neutral pH systems due to their fast degradation in basic media via a dealkylation process driven by a nucleophilic attack of hydroxide. In this study, a family of viologen-based anolytes suitable for alkaline systems is introduced, demonstrating that properly designed viologens can also be used in alkaline conditions. A variety of *N*-aryl viologens are prepared and characterized, showing that the dealkylation process is prevented by bonding an aryl group directly to the N-atom of the bipyridine core. Pairing **B-2,5-DHPV** for the anolyte and K_4_Fe­(CN)_6_ for the catholyte, a full alkaline AORFB having a nominal cell voltage at 0.98 V maintains stable capacity over 1400 continuous cycles with nearly 0.03%·h^–1^ capacity decay, which is a very acceptable value for a viologen in an alkaline medium. Our results enable the broadening of the range of viable organic anolytes for alkaline AORFBs.

## Graphical abstract

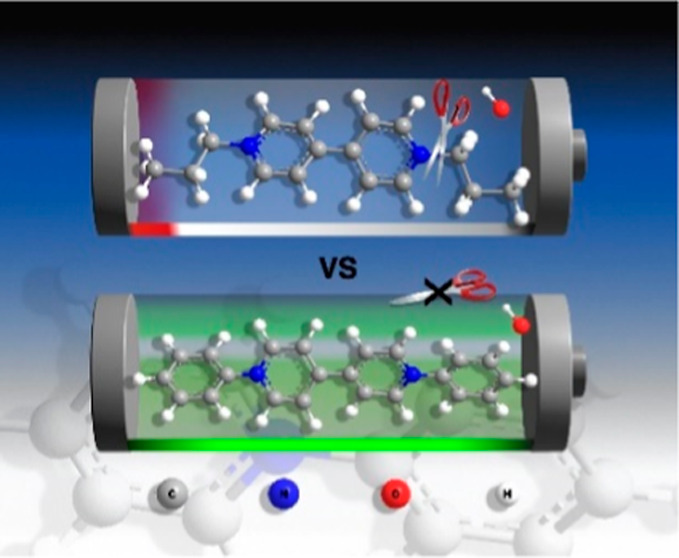

## 1 Introduction

Developing large-scale, safe, and cost-effective energy storage systems is essential to harness renewable energy effectively.^1–3^ Aqueous redox flow batteries (ARFBs) stand out as highly promising candidates for storing energy from intermittent sources such as solar and wind power due to their nonflammable electrolytes and the ability to independently scale energy and power. While vanadium redox flow batteries are the most mature ARFBs, they face economic and resource challenges due to their reliance on critical materials.^4^ Aqueous organic redox flow batteries (AORFBs) represent a compelling alternative, using diverse, cost-effective, and structurally tunable organic molecules.^5^ Viologens are particularly attractive as anolytes in neutral AORFBs for their adequate redox kinetics, suitable redox potential, and feasible synthesis.^6,7^ Viologen derivatives previously reported as anolytes in AORFBs exclusively present alkyl groups as substituents for the quaternization of nitrogen atoms in the 4,4′-bipyridine core.^8–13^ Figure 1 shows the structures of the most representative derivatives described in the literature for these applications.

**Figure 1:**
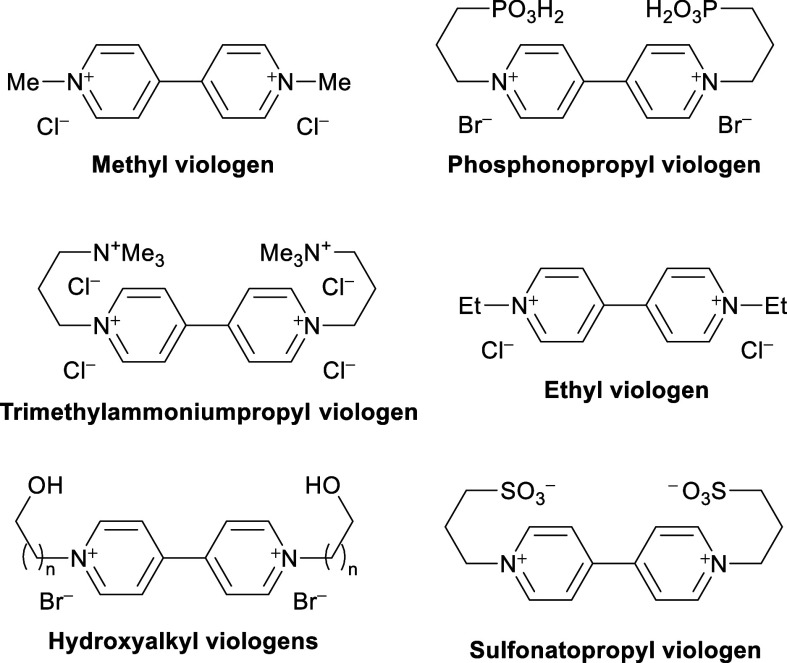
Representative selection of viologen derivatives described in the literature as anolytes for AORFBs which operate strictly and exclusively in neutral/nonalkaline media.

Therefore, the operating conditions for these batteries should be strictly and exclusively near neutral media, as viologens are reported to be unstable in alkaline media by several authors.^14–17^ However, alkaline battery systems present significant advantages compared to neutral media including elevated conductivity, increased power output, the ability to easily address Faradaic imbalance, and greater stability of pH level (the pH value fluctuates easily in neutral pH due to the logarithmic ratio between pH and OH^–^ concentration).^18–23^ Despite these advantages, these systems have been primarily limited to the use of quinone, phenazine, or fluorenone derivatives as anolytes for AORFBs.^24–26^ Therefore, there is great interest in developing organic electroactive materials suitable for alkaline systems to enable AORFBs to capitalize on these advantageous features. In this context, our research group has studied the chemical stability of these electroactive species in alkaline media, analyzing the decomposition process of 1,1′-bis-3-sulfonatopropyl viologen (**(SPr)**_**2**_ **V**), which can be considered as the state-of-the-art derivative under alkaline conditions. It is postulated that this degradation involves a dealkylation process promoted by the nucleophilic attack of the hydroxide anion on C­(sp^3^) directly bonded to the nitrogen atom (Scheme 1A). Through molecular engineering, we showed that this degradation process can be significantly slowed down by increasing the steric hindrance around the site susceptible to nucleophilic attack (Scheme 1B).^27^ Unfortunately, this strategy does not completely prevent degradation but only slows down the process under mild alkaline conditions (pH 9–11). As with viologens, other electroactive species for redox flow batteries, such as anthraquinones, undergo decomposition by nucleophilic substitution under alkaline conditions.^28^

**Scheme 1:**
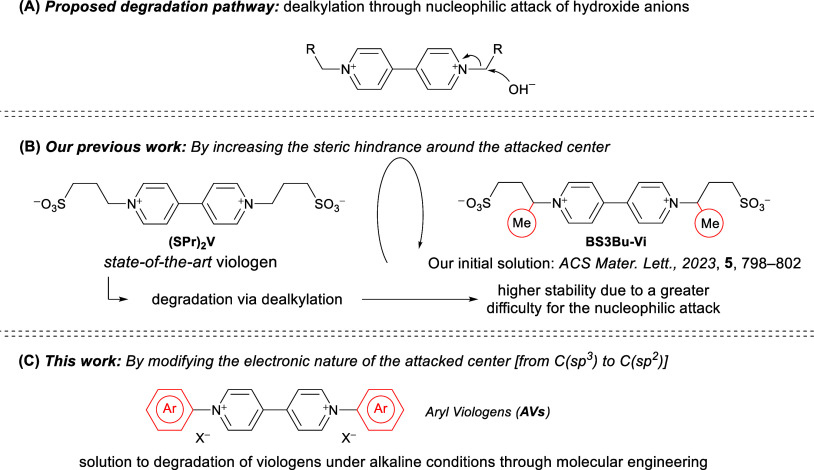
(A) Proposed Degradation Pathway for Viologen-Based Anolytes. (B) Our Previous Work and Our Initial Solution to Improve the Chemical Stability of Viologens under Alkaline Conditions. (C) Our Proposal to Solve the Problem

To mitigate this undesired pathway, which is associated with capacity decay, Aziz et al. addressed the problem for anthraquinones by replacing carbon–heteroatom by carbon–carbon bonds between redox centers and lateral chains, considering that C–C bonds are more chemically resistant against nucleophilic attack than carbon–heteroatom bonds like C–O (S, N).^29^ However, this elegant strategy is not suitable for viologens because the presence of nitrogen atoms is required for the electroactivity of the compound as they are essential components of the redox core in the structure of this family of electroactive species. For these reasons, a new approach must be devised if viologen derivatives are to be used in alkaline AORFBs. In this work, we propose a new family of viologen-based anolytes capable of withstanding strong alkaline conditions due to their increased chemical stability against nucleophilic attack. In this context, we envisioned that complete inhibition of the degradation pathway, based on the nucleophilic attack of hydroxide anions on the C atoms bonded to the N atoms of the bipyridinium core, could be achieved by changing the electronic nature of these C atoms. Thus, we propose the introduction of aryl groups as *N*-substituents to completely prevent the nucleophilic attack of hydroxide anions (Scheme 1C). Given the higher electronegativity of C­(sp^2^), compared to C­(sp^3^), we predicted that viologen decomposition through the cleavage of the C–N bond would be greatly disfavored for C­(sp^2^)–N bonds. According to this proposal, aryl viologens, derived from a bipyridine scaffold substituted with aryl groups at the N atoms, would exhibit higher stability under alkaline conditions. It should be noted that the presence of aromatic groups in the viologen molecule could reduce the solubility in aqueous media. Thus, further modification of the structure by introducing hydrophilic groups is necessary to improve the overall performance.

## 2 Experimental Section

### 2.1 Materials

All common reagents and solvents were purchased from Aldrich or Alfa-Aesar and used as received without further purification.

### 2.2 NMR Measurements

NMR spectra were measured on a Bruker Avance III HD 300 MHz spectrometer. ^1^H NMR: splitting pattern abbreviations are s, singlet; d, doublet; t, triplet; q, quartet; dd, double doublet; ddd, doublets of doublets of doublets; ddt, double doublet of triplets; dt, doublet of triplets; dq, doublet of quartets; td, triplet of doublets; qd, quartet of doublets; p, pentuplet; h, sextet; hept, heptet; m, multiplet; b, broad; a, apparent; the chemical shifts are reported in ppm using the residual solvent peak as a reference. ^13^C NMR spectra were recorded at 75.4 MHz using broad-band proton decoupling, and chemical shifts are reported in ppm using adequate solvent peaks as internal reference (CH_3_OH: 49.50), and the multiplicities were determined by DEPT experiments.

### 2.3 pH Measurement

pH measurements were determined using an Accumet AB150 device which allows us to record pH values at different times.

### 2.4 Cyclic Voltammetry

Cyclic voltammetry studies were performed using an Autolab PGSTAT12 instrument (Methrom-Autolab, The Netherlands) with NOVA 2.1.3 software. A three-electrode cell was employed using a polished glassy carbon working electrode (*A*_electrode_ = 7 mm^2^) and a Pt wire counter electrode (99% purity), and the Ag/AgCl (3 M KCl) electrode was used as an aqueous reference electrode.

### 2.5 Static Battery Assembly

A static cell was designed using SketchUp software and manufactured using an ultraviolet (UV) liquid-crystal-display-based stereolithography 3D printer (Photon Mono SE, Anycubic) and a commercial clear resin (Anycubic). A filter-pressed static cell using expanded graphite (SGL Carbon), graphite felt (SGL Carbon), and Nafion 212 (Ion Power) as the current collector, electrode, and ion-selective membrane, respectively, was used. The projected area of the cell was 3 cm^2^ (internal volume ≈0.38 mL). Galvanostatic charge–discharge measurements were performed by using a Neware BTS battery testing system CT-40087-5 V6A-S1. The batteries were charged at 5 mA·cm^–2^ with voltage limits at 1.2 V. Thereafter, the batteries were discharged at 5 mA·cm^–2^ with a voltage limit of 0.5 V. General conditions: the battery was filled using 2 mL of the anolyte with 0.2 M viologen in 1.0 M KCl and 0.8 M KOH and 2 mL of the catholyte with 0.3 M K_4_Fe­(CN)_6_ in 1.0 M KCl and 0.8 M KOH. All electrolytes were prepared with deionized water, and both were purged with argon prior to use.

### 2.6 Flow Battery Assembly

Filter-pressed flow cells using Nafion 212 and graphite felt as the ion-selective membranes and electrodes were used in this study. The projected area of the cell was 9 cm^2^. The flow rate was fixed at about 50 mL·min^–1^. Galvanostatic and potentiostatic (constant current, followed by constant voltage protocol (CC–CV)) charge–discharge measurements were realized using a Neware BTS battery testing system CT-40087-5 V6A-S1. The batteries were charged at 30 mA·cm^–2^ with voltage limits at 1.2 V. Thereafter, the batteries were discharged at −30 mA·cm^–2^ with a voltage limit of 0.5 V under Ar atmosphere. General conditions: anolyte (12 mL), 0.2 M viologen in 1.0 M KCl, and 0.8 M KOH; catholyte (45 mL), 0.3 M K_4_Fe­(CN)_6_ in 1.0 M KCl, and 0.8 M KOH.

### 2.7 Solubility Test

The solubility of viologen **B-2,5-DHPV** in 1 M KOH was determined by using UV–vis spectrophotometry at 240 nm. A calibration curve was first established by measuring the absorbance of a series of standard **B-2,5-DHPV** solutions with known concentrations. To obtain the test sample, **B-2,5-DHPV** was dissolved in 1 M KOH until saturation was reached, followed by the removal of any undissolved solid through filtration. The saturated solution was subsequently diluted to bring a solution with absorbance within the linear range of the calibration curve, from which the concentration was quantified.

## 3 Results and Discussion

In order to prove our proposal, we considered to use the Zincke reaction that allows the synthesis of a family of aryl viologen derivatives (**AVs**) bearing different aryl rings attached to the bipyridinium core and functionalized at several positions with acidic functional groups, such as free carboxylic acids.^30–32^ The Zincke salt from 4,4′-bipyridine (**1**) was subjected to the Zincke reaction, an overall amine exchange process to prepare *N*-alkyl or *N*-arylpyridinium salts, using different ester-functionalized anilines to yield viologen derivatives **2a**–**e** (Scheme 2A). After the completion of the reaction and without further purification, acid hydrolysis led to the formation of the corresponding bis­(carboxyphenyl) viologen derivatives **AV-3a–e**. On the other hand, an easy and straightforward access to 2,5-dihydroxyphenyl viologen **B-2,5-DHPV** (**AV-3f**) was achieved through the Michael addition of both pyridyl moieties to *p*-benzoquinone, allowing access to an aryl viologen functionalized with hydroxyl groups (Scheme 2B).^33^ Detailed synthetic procedures and product characterization are provided in the Supporting Information.

**Scheme 2:**
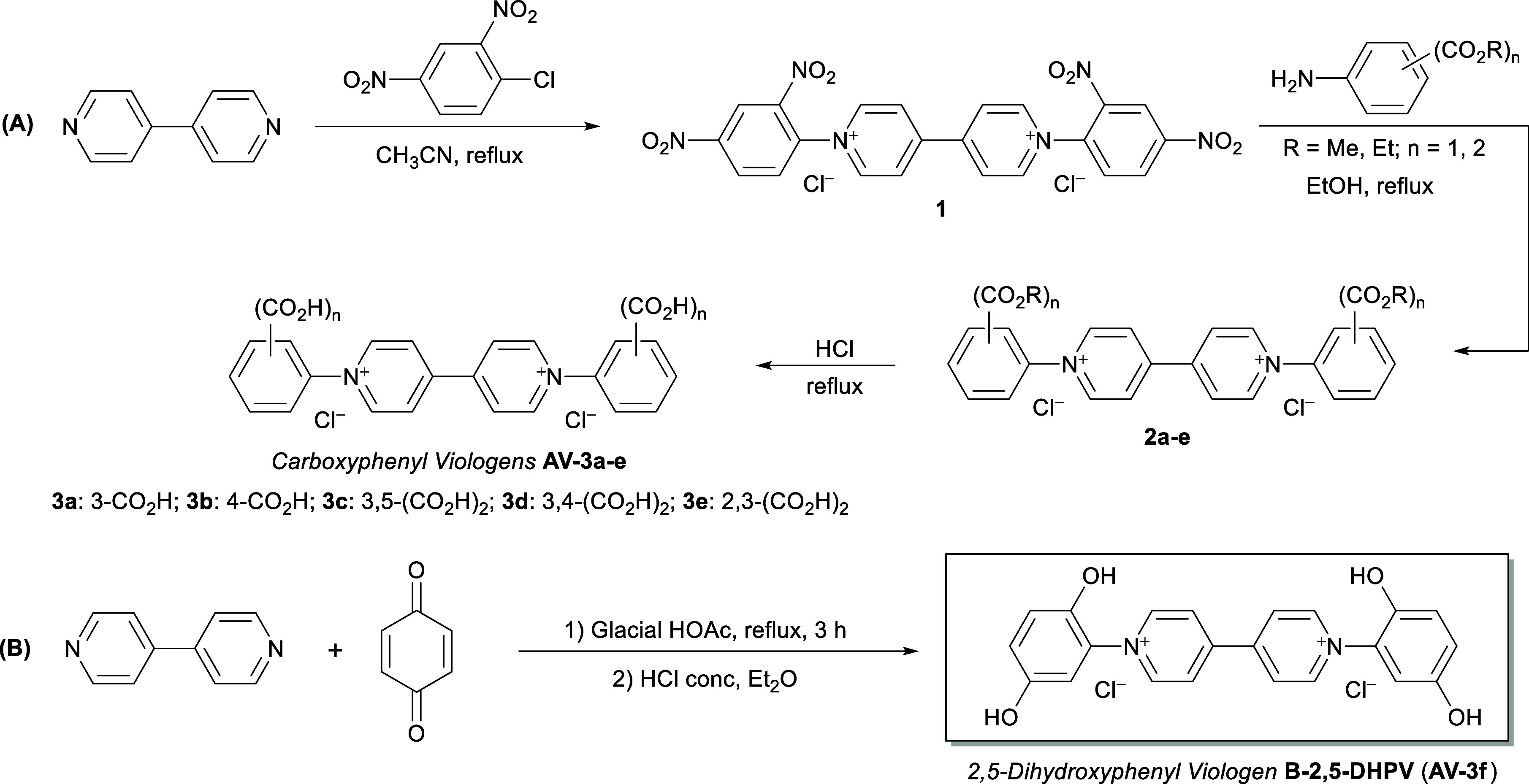
Synthetic Routes for the Preparation of (A) Carboxyphenyl Viologen Derivatives **AV-3a–e** and (B) Hydroxyphenyl Viologen Derivative **AV-3f**

A map of redox potentials for the synthesized aryl viologens **AV-3a–f** at pH 14 along with phenyl viologen (**PV**)^34^ is shown in Figure 2 (the cyclic voltammetry result of each of the prepared compounds is provided in the Supporting Information; see Section S2). Regarding the electrochemical properties of these viologen derivatives, it should be noted that all of them are electroactive compounds that exhibit good reversible behavior in cyclic voltammetry in alkaline media. Furthermore, the redox potential could be modulated up to 400 mV, depending on the nature of the substituents on the aryl groups. The withdrawing and donating characters of the functional groups have been reported to influence the redox potential of the molecules, so it is anticipated that the redox potential and thus the cell voltage can be slightly tuned by selecting the proper groups.^35^ Therefore, as expected from the literature, viologen **AV-3f** with electron-donating substituents such as hydroxyls presents a redox potential more negative than that of viologens **AV-3a–e** substituted with electron-withdrawing groups such as carboxyls. These results indicate the high potential of aryl viologen derivatives as a promising new family of viologen-based anolytes for AORFBs. The viologen derivative **B-2,5-DHPV** (**AV-3f**) was chosen for investigation in full cells due to its easily accessible synthesis, low cost of starting materials enabling mass production, and suitable redox potential.

**Figure 2:**
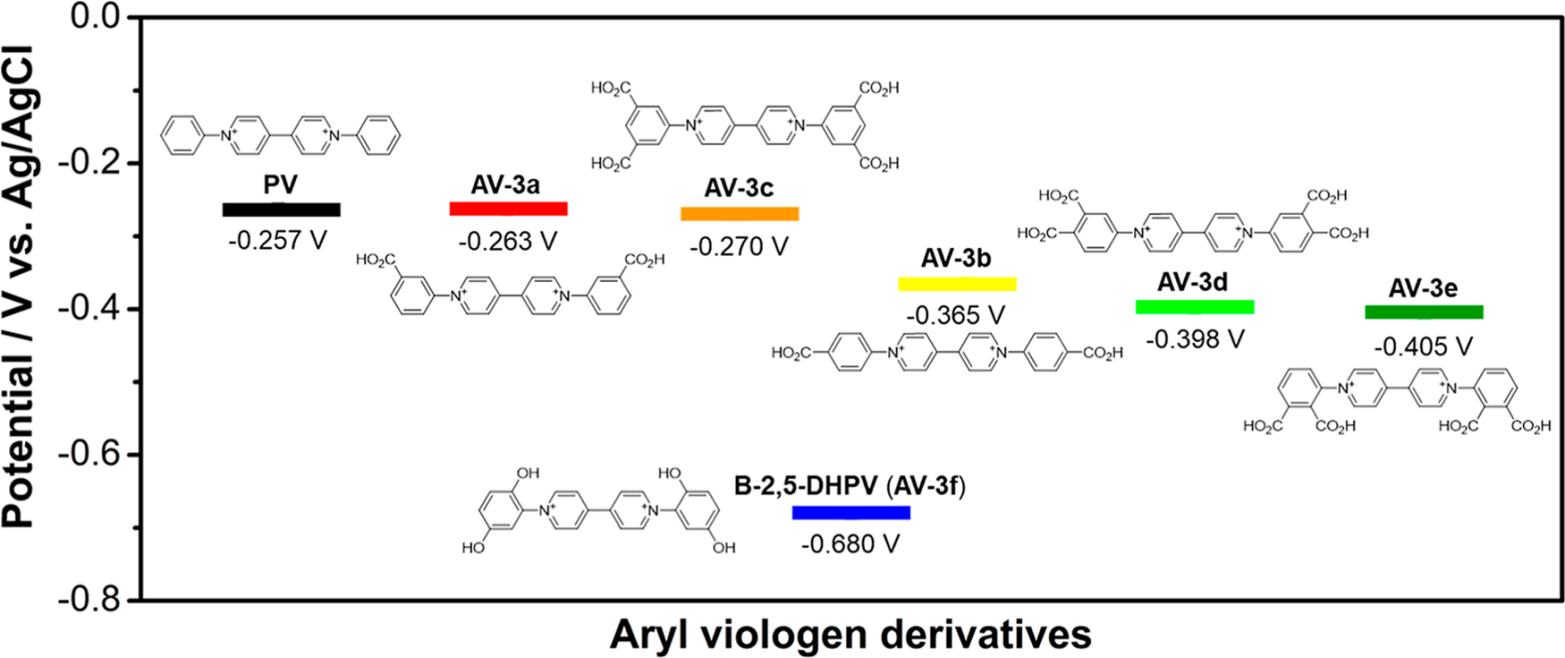
Redox potentials of aryl viologen derivatives (**AVs**) at pH 14 including phenyl viologen **PV**.

Before using viologen **B-2,5-DHPV** in redox flow batteries, its chemical stability in alkaline media was explored by using ^1^H NMR. Figure 3 shows the ^1^H NMR spectra of the state-of-the-art viologen **(SPr)**_**2**_ **V**, viologen **BS3Bu-Vi**,^27^ viologen **(DBPPy)­Cl**_**4**_,^36^ and the aryl viologen **B-2,5-DHPV** before and after being exposed to pH 14 by the addition of KOH. The upper ^1^H NMR spectrum in Figure 3A reveals the expected decomposition of the viologen **(SPr)**_**2**_ **V**, which degrades quickly in alkaline media after ca. 5 min of exposure. The same decomposition process has also been observed in previous studies.^15–18,27^ Analogously, **BS3Bu-Vi** and **(DBPPy)­Cl**_**4**_ (Figure 3B,C), two alkyl viologens designed to present enhanced stability toward the nucleophilic attack of hydroxide anions by increasing steric effects, suffered the same fast decomposition processes.

**Figure 3:**
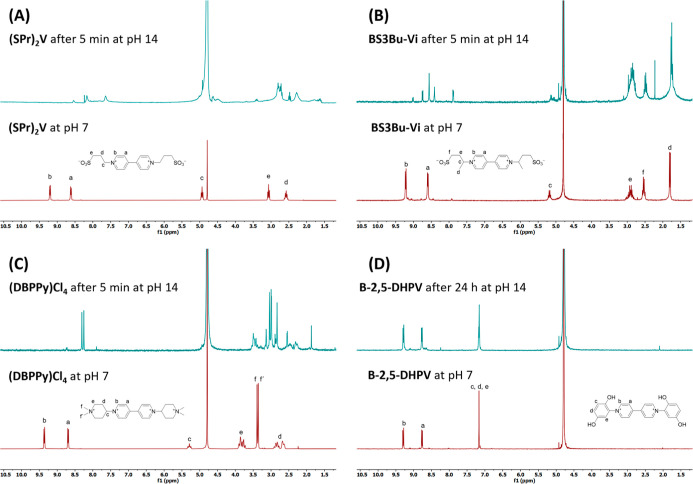
Chemical stability studies at pH 14 of the state-of-the-art viologen **(SPr)**_**2**_ **V**, two viologens with increased steric hindrance (**BS3Bu-Vi** and **(DBPPy)­Cl**_**4**_), and aryl viologen **B-2,5-DHPV** (**AV-3f**). (A) Stacked ^1^H NMR spectra of **(SPr)**_**2**_ **V** at pH 14 for 5 min (blue) and at pH 7 (red). (B) Stacked ^1^H NMR spectra of **BS3Bu-Vi** at pH 14 for 5 min (blue) and at pH 7 (red). (C) Stacked ^1^H NMR spectra of **(DBPPy)­Cl**_**4**_ at pH 14 for 5 min (blue) and at pH 7 (red). (D) Stacked ^1^H NMR spectra of **B-2,5-DHPV** at pH 14 for 24 h (blue) and at pH 7 (red). The experiments were performed in D_2_O as a solvent under N_2_ atmosphere, and the pH was adjusted using KOH and DCl. The pH was brought back to neutral after exposure to alkaline condition for better comparison.

In contrast, the aryl viologen **B-2,5-DHPV** remains chemically stable even after 24 h of exposure at pH 14, with the spectra recorded before and after this time being identical, which supports its excellent chemical stability (Figure 3D). These results confirm that changing the electronic nature of the carbon atoms, from C­(sp^3^) (alkyl chain) to C­(sp^2^) (aryl group), directly bonded to the N atoms of the bipyridinium core allows for the complete inhibition of degradation by cleavage of the *N*-substituents via nucleophilic attack by the hydroxide anions under alkaline conditions. This highlights that aryl viologen derivatives are remarkably more stable under basic pH than alkyl viologen derivatives.

Initially, a static battery test was conducted using viologen **B-2,5-DHPV** as the anolyte to study its cyclability in alkaline conditions, employing K_4_Fe­(CN)_6_ as the catholyte (Figure 4). As the solubility of **B-2,5-DHPV** in 1 M KOH was determined to be 0.29 M, the concentration of **B-2,5-DHPV** in the anolyte was set to 0.2 M to ensure complete dissolution. The anolyte solution with a concentration of 0.2 M **B-2,5-DHPV** in 1 M KCl and 0.8 M KOH reached a pH value of 14. The simplicity of static cells facilitates the evaluation of the intrinsic stability of the compound.^37^ The theoretical cell voltage of **B-2,5-DHPV**//K_4_Fe­(CN)_6_ is 0.98 V (Figure 4A), a value slightly superior to the **(SPr)**_**2**_ **V**//K_4_Fe­(CN)_6_ battery voltage, which enables a 0.81 V cell (Figure 4B). The results reveal an exceptional behavior due to its great cyclability over more than 2000 cycles (14 days), excellent Coulombic efficiency, and low capacity fading throughout the battery performance (0.024%·h^–1^), highlighting the potential utility of **B-2,5-DHPV** as an anolyte for alkaline batteries (Figure 4C,D).

**Figure 4:**
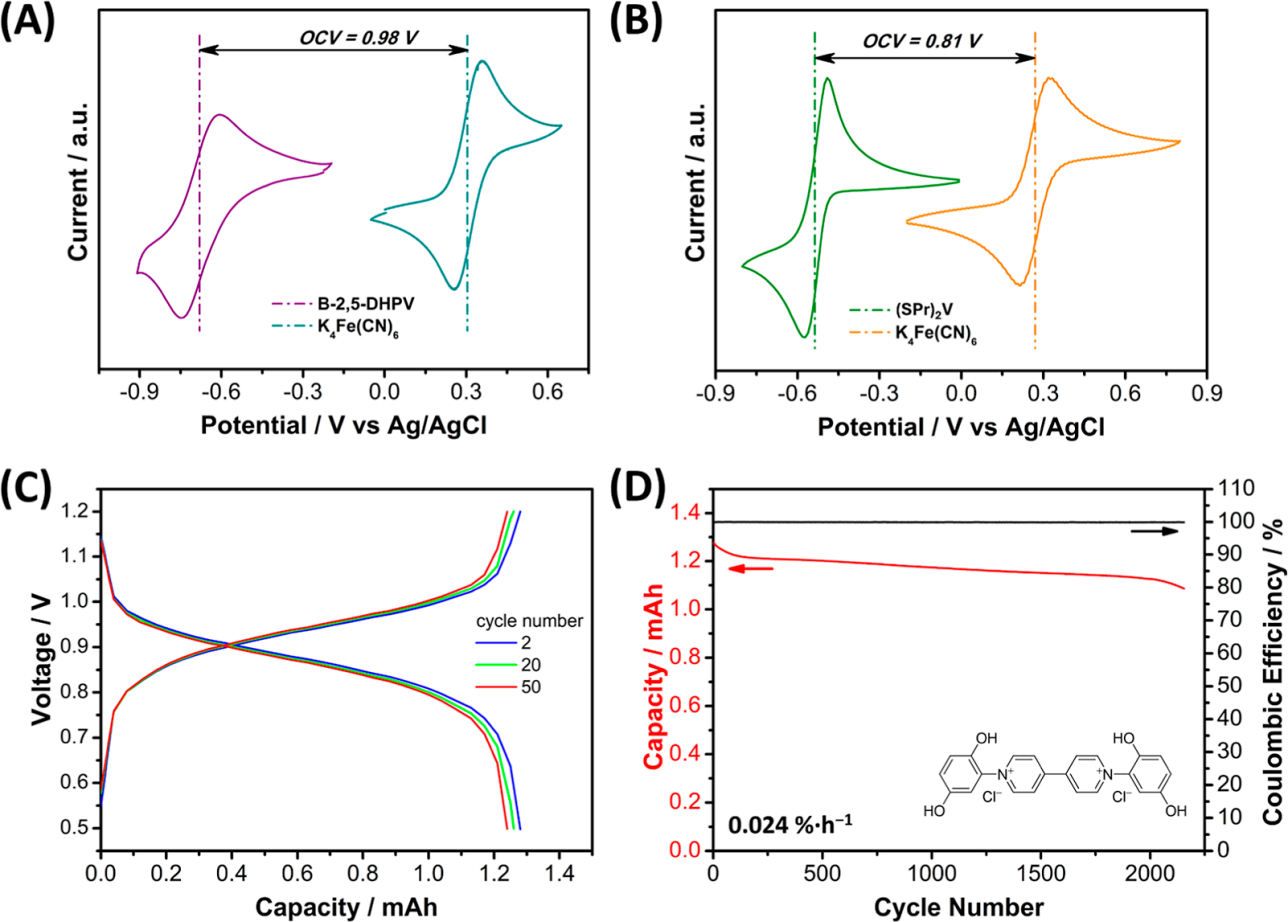
(A) CV curves for **B-2,5-DHPV** and K_4_Fe­(CN)_6_ in 1 M KCl and 0.8 M KOH. The equilibrium potential of **B-2,5-DHPV**//K_4_Fe­(CN)_6_ is 0.98 V. (B) CV curves for **(SPr)**_**2**_ **V** and K_4_Fe­(CN)_6_ in 1 M KCl. The equilibrium potential of **(SPr)**_**2**_ **V**//K_4_Fe­(CN)_6_ is 0.81 V. (C) Charge and discharge profiles for the **B-2,5-DHPV**//K_4_Fe­(CN)_6_ alkaline static battery for cycle numbers 2, 20, and 50. (D) Evolution of the charge capacity and Coulombic efficiency of the static cell upon cycling having **B-2,5-DHPV**//K_4_Fe­(CN)_6_ in 1 M KCl and 0.8 M KOH (14 days).

Finally, a full flow cell was assembled using viologen **B-2,5-DHPV** and K_4_Fe­(CN)_6_ as an anolyte and catholyte, respectively (Figure 5). The cell demonstrated stable cycling at 30 mA·cm^–2^ for 21 days (1425 cycles) (Figure 5B), with a 0.025%·h^–1^ of capacity decay over the last 1000 cycles, which is significantly better than the 0.45%·h^–1^ reported by our group for the state-of-the-art viologen **(SPr)**_**2**_ **V** in neutral media under comparable conditions.^27^ Notably, this capacity retention value in alkaline media is comparable to some of the values reported for viologen-based anolytes in neutral pH.^23,38^ Since the capacity of the catholyte is 1.5 times that of the anolyte and the anolyte crossover rate is negligible (as confirmed by permeability experiments; see Section S3 of Supporting Information), the capacity fade is primarily attributed to the decomposition of the electroactive material in the anolyte. Furthermore, the permeability presented by the aryl viologen **B-2,5-DHPV** was several orders of magnitude lower than that corresponding to the state-of-the-art viologen **(SPr)**_**2**_ **V** under identical experimental conditions. The permeability values of **B-2,5-DHPV** and **(SPr)**_**2**_ **V** were 6.35 × 10^–11^ cm^2^·s^–1^ and 1.07 × 10^–7^ cm^2^·s^–1^, respectively (see Section S3 of Supporting Information). This significant difference is likely due to the larger size of the aryl viologen derivatives. Importantly, the diffusion coefficient value of 2.98 × 10^–7^ cm^2^·s^–1^ for aryl viologen **B-2,5-DHPV** (see Section S4 of Supporting Information) was not significantly affected by the increased size, as the value is of the same order of magnitude as those reported for organic anolytes in AORFBs.^39–42^ Considering that no appreciable degradation occurs from the nucleophilic attack of hydroxyls on the aryl viologen **B-2,5-DHPV**, and that the system exhibits negligible crossover, we initially attribute the observed capacity decrease in AORFB (Figure 5) to the formation of π–π aggregates, as has been reported for alkyl viologens when operating in a glovebox. In addition, note that the synthesized **B-2,5-DHPV** product has a purity of approximately 95% and contains ≈4% hydration molecules, as experimentally determined. Taking these factors into account, the theoretical capacity, assuming a one-electron transfer, is estimated to be 0.059 Ah. Under these considerations, the AORFB shown in Figure 5 reaches an initial capacity of 93% of the theoretical capacity.

**Figure 5:**
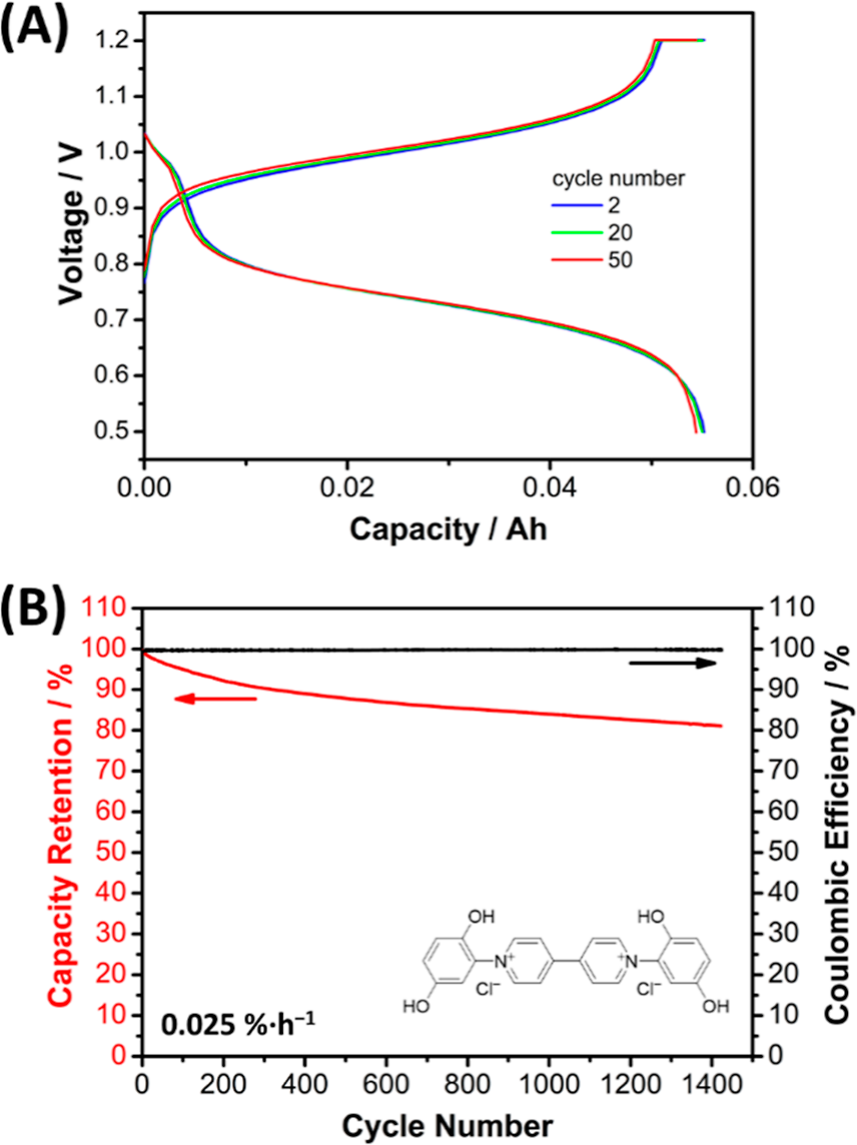
Performance of the full flow cell having 12 mL of 0.2 M **B-2,5-DHPV**//45 mL 0.3 M K_4_Fe­(CN)_6_ in 1 M KCl and 0.8 M KOH (pH 14) using Ar overpressure in the negative compartment with 0.2 M of **B-2,5-DHPV** as the anolyte in an alkaline AORFB. (A) Charge and discharge profiles for **B-2,5-DHPV**//K_4_Fe­(CN)_6_ alkaline AORFB for cycle numbers 2, 20, and 50. (B) Evolution of the charge capacity of the flow cell upon cycling (21 days). The cell was first charged/discharged at 30 mA·cm^–2^ until voltages reached 1.2 or 0.5 V and then was held at these voltages until the current density dropped to 1 mA·cm^–2^.

## 4 Conclusions

In summary, to the best of our knowledge, this is the first report demonstrating the feasibility of using a viologen derivative as the anolyte in alkaline flow batteries, capitalizing on the advantages offered by such conditions. Through the use of molecular engineering, it is possible to improve the stability of electroactive species. It has been proven that modifying the electronic nature of the atom involved in the degradation of viologens in an alkaline medium is more satisfactory than solutions based on steric hindrance previously described in the literature. These results highlight the capability of viologens to function effectively in strong alkaline environments, contrary to previous assumptions, thus expanding the range of organic compounds suitable for anolyte materials in such systems. This finding paves the way for the development of new viologen-based electroactive materials in the field of alkaline organic redox flow batteries.

## Supplementary Material



## Notes

Detailed experimental synthetic procedures; ^1^H and ^13^C NMR spectra and cyclic voltammograms for all compounds; UV–Vis spectra for crossover evaluation; and cyclic voltammograms for the diffusion coefficient determination (PDF)
