# Supplementary material for: Aryl Viologens: Unprecedented Stability of Viologen-Derivatives as Anolytes for Alkaline Redox Flow Batteries
Source: ACS Appl Energy Mater. 2025 Nov 3;8(22):16524–31. doi: 10.1021/acsaem.5c02255 (PMC12661920; doi:10.1021/acsaem.5c02255)
Supplement: Supplementary file 1 [file ae5c02255_si_001.pdf]

Supporting Information

Aryl Viologens: Unprecedented Stability of Viologen-Derivatives as Anolytes for Alkaline Redox Flow Batteries

Rubén Rubio-Presa,*[a,b] Edgar Ventosa *[a,b] and Roberto Sanz *[a]

[a]*Department of Chemistry, Faculty of Science, University of Burgos, Pza. Misael Bañuelos s/n, 09001-Burgos (Spain).*

[b]*International Research Center in Critical Raw Materials-ICCRAM, Universidad de Burgos, Pza. Misael Bañuelos s/n, E-09001, Burgos (Spain).*

E-mail: rrpresa@ubu.es; eventosa@ubu.es; rsd@ubu.es

Index

General Methods S2

Section S1. Synthesis and Characterization S4

Section S2. Electrochemical Characterization S29

Section S3. Crossover Evaluation S30

Section S4. Estimation of Diffusion Coefficient S32

Section S5. Evolution of the Capacity for B-2,5-DHPV // $K_4Fe(CN)_6$ S33

References S34

General Methods

Materials.

All common reagents and solvents were purchased from Aldrich or Alfa-Aesar and used as received without further purification.

NMR measurements.

NMR spectra were measured on Bruker Avance III HD 300 MHz spectrometer. ^{1}H NMR: splitting pattern abbreviations are: s, singlet; d, doublet; t, triplet; q, quartet; dd, double doublet; ddd, doublets of doublets of doublets; ddt, double doublet of triplets; dt, doublet of triplets; dq, doublet of quartets; td, triplet of doublets; qd, quartet of doublets; p, pentuplet; h, sextet; hept, heptet; m, multiplet; b, broad; a, apparent; the chemical shifts are reported in ppm using residual solvent peak as reference. ^{13}C NMR spectra were recorded at 75.4 MHz using broadband proton decoupling and chemical shifts are reported in ppm using adequate solvent peaks as internal reference (CH_3OH: 49.50) and the multiplicities were determined by DEPT experiments.

Melting points determination.

Melting points were measured on a Gallenkamp apparatus using open capillary tubes and are uncorrected.

pH measurement.

pH measurements were determined using an Accument AB150 device which allows to record pH values at different times.

Cyclic voltammetries.

Cyclic voltammetry studies were performed using an Autolab PGSTAT12 (Methrom-Autolab, The Netherlands) with NOVA 2.1.3 software. A three-electrode cell was employed using a

polished glassy carbon working electrode ($A_{electrode}$ = 7 mm^2), a Pt wire counter electrode (99% purity) and the Ag/AgCl (3 M KCl) electrode was used as aqueous reference electrode.

Static batteries.

The static cell was designed using the SketchUp software and manufactured using an ultraviolet (UV) liquid-Crystal-display-based stereolithography 3D printer (Photon Mono SE, Anycubic) and a commercial clear resin (Anycubic). After the cleaning procedure (with 70% isopropanol solution), the printed pieces were assembled. A filter-pressed static cell using expanded graphite (SGL Carbon), graphite felt (SGL Carbon), and Nafion 212 (Ion Power) as current collector, electrode, and ion-selective membrane, respectively, was used. The projected area of the cell was 3 cm^2 (internal volume ≈0.38 mL). For further details on the components and assembly of the static battery, see reference 37 in the main text. Galvanostatic charge–discharge measurements were performed using a Neware BTS battery testing system CT-40087-5V6A-S1. The batteries were charged at 5 $mA \cdot cm^{-2}$ with voltage limits at 1.2 V. Thereafter, batteries were discharge at 5 $mA \cdot cm^{-2}$ with a voltage limit of 0.5 V. General conditions: The battery was filled using 2 mL for the anolyte with 0.2 M viologen in 1.0 M KCl and 0.8 M KOH; and 2 mL for the catholyte with 0.3 M $K_4Fe(CN)_6$ in 1.0 M KCl and 0.8 M KOH. All electrolytes were prepared with deionized water and both were purged with argon prior to use.

Flow batteries.

Filter-pressed flow cells using Nafion® 212 and graphite felt as the ion selective membrane and electrodes were used in this study. The projected area of the cell was 9 cm^2. The flow rate was fixed at ca. 50 $mL \cdot min^{-1}$. Galvanostatic and potentiostatic (constant current followed by constant voltage protocol (CC-CV)) charge-discharge measurements were realized using a Neware BTS battery testing system CT-40087-5V6A-S1. The batteries were charged at 30 $mA \cdot cm^{-2}$ with voltage limits at 1.2 V. Thereafter, batteries were discharge at −30 $mA \cdot cm^{-2}$ with a voltage limit

of 0.5 V under Ar atmosphere. General conditions: anolyte (12 mL), 0.2 M viologen in 1.0 M KCl and 0.8 M KOH; catholyte (45 mL), 0.3 M $K_4Fe(CN)_6$ in 1.0 M KCl and 0.8 M KOH.

UV-Vis spectra.

UV-Vis spectra were acquired on a Varian Cary 50 Conc UV-Vis spectrophotometer using a Quartz SUPRASIL cell with a 10 mm light path.

Section S1. Synthesis and Characterization

Synthesis and Characterization Data of Starting Materials

Synthesis of 1,1′-bis(2,4-dinitrophenyl)-[4,4'-bipyridine]-1,1′-diium dichloride (Zincke salt):

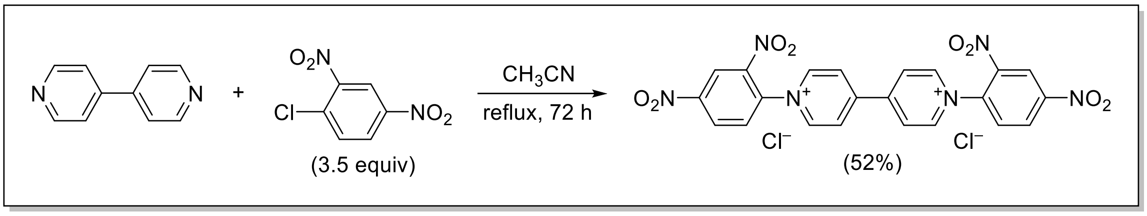

4,4′-Bipyridine Zincke salt was prepared according to a reported method.[1]

A solution of 4,4′-bipyridine (3.2 g, 20.5 mmol) and 1-chloro-2,4-dinitrobenzene (14.2 g, 70 mmol), in anhydrous acetonitrile (60 mL) was heated under reflux for 72 h under an inert nitrogen atmosphere. The reaction mixture was diluted with acetonitrile (50 mL) and filtered off. The filtered cake was refluxed with EtOH (350 mL). After cooling to room temperature, a greyish white solid was filtered off, washed with EtOH and dried under vacuum. The crude product was purified by recrystallization form MeOH/EtOAc.

The spectral data of the compound are in good agreement with the reported data.[2]

1,1′-Bis(2,4-dinitrophenyl)-[4,4′-bipyridine]-1,1′-diium dichloride: Yielded (6 g, 52%) as a greyish solid.

^{1}H NMR (300 MHz, D_2O): δ = 9.57 (d, *J* = 7.1 Hz, 4H, Ar*H*), 9.46 (d, *J* = 2.5 Hz, 2H, Ar*H*), 9.05−8.99 (m, 6H, Ar*H*), 8.40 (d, *J* = 8.7 Hz, 2H, Ar*H*).

^{1}H-NMR spectrum.

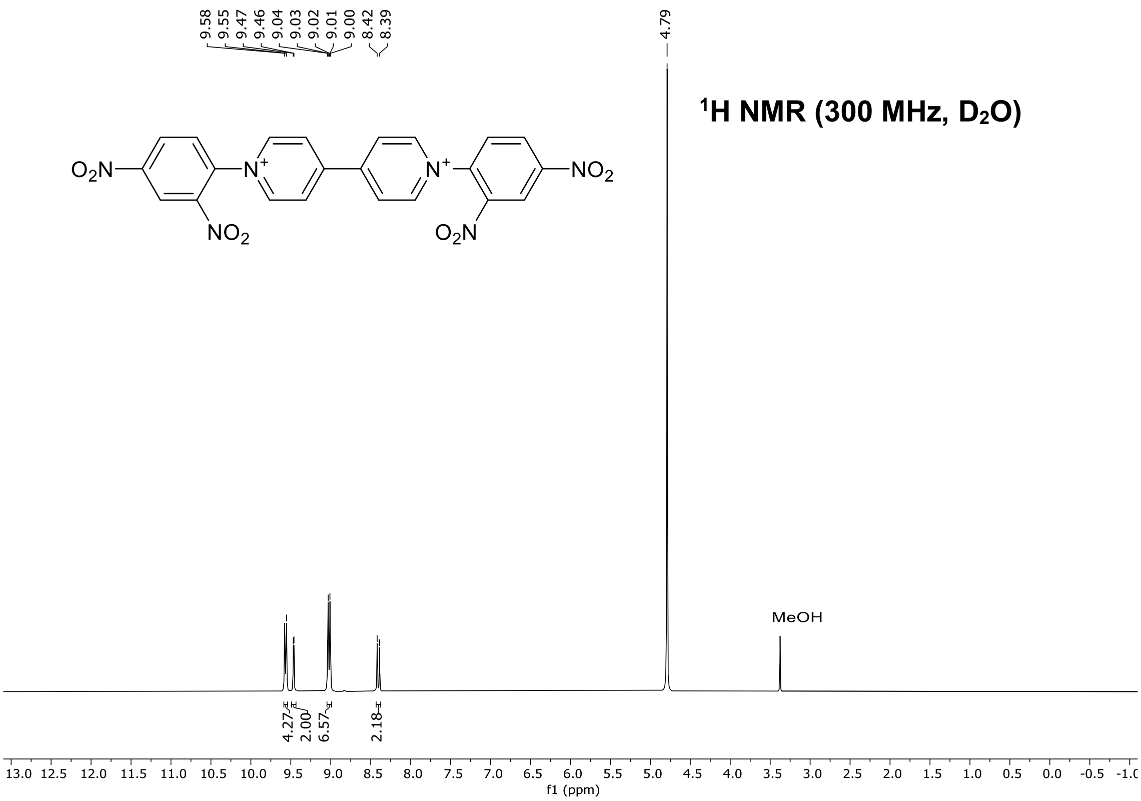

Synthesis of starting anilines:

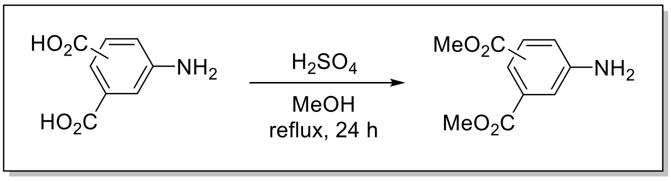

General procedure: A mixture of the corresponding aniline (39 mmol), MeOH (80 mL) and H_2SO_4 (16 mL) was heated under reflux for 12−24 h. Then, the reaction mixture was concentrated under vacuum. The residue was dissolved in cold H_2O (40 mL) and the resulting solution was neutralized with a saturated aqueous solution of $NaHCO_3$, while maintaining the temperature below 10 ºC. Next, the mixture was extracted with EtOAc (4 × 75 mL). The combined organic phases were dried over anhydrous Na_2SO_4 and concentrated under reduced pressure to give the corresponding crude product.

Dimethyl 5-aminoisophthalate:[3] Yielded (6.85 g, 84%) as a brownish solid. After 24 h reaction time, the product was obtained in pure form without further purification.

^{1}H NMR (300 MHz, DMSO-d_6): δ = 7.64 (t, *J* = 1.6 Hz, 1H, Ar*H*), 7.41 (d, *J* = 1.6 Hz, 2H, Ar*H*), 5.74 (bs, 2H, N*H*$_2$), 3.83 (s, 6H, OC*H*$_3$).

^{1}H-NMR spectrum.

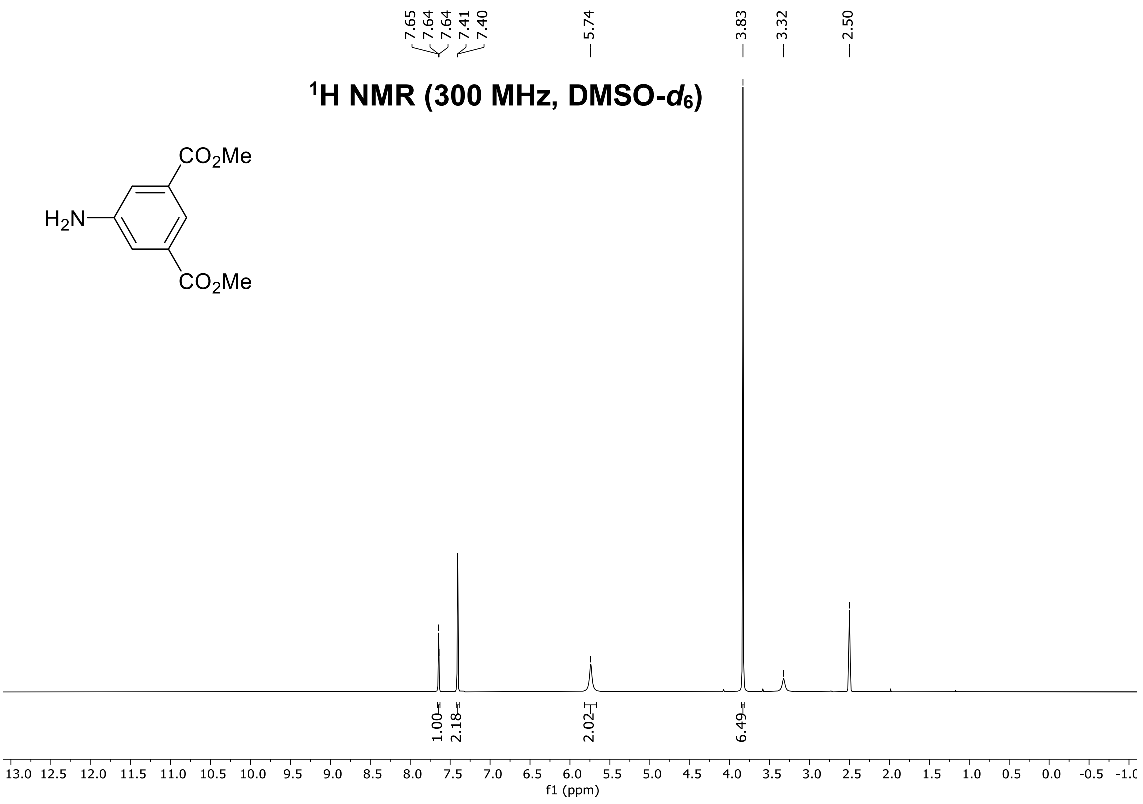

Dimethyl 4-aminophthalate:[4] Yielded (6.77 g, 83%) as a yellowish solid. After 24 h reaction time, the product was obtained in pure form without further purification.

^{1}H NMR (300 MHz, $CDCl_3$): δ = 7.71 (dd, *J* = 8.4 and 0.5 Hz, 1H, Ar*H*), 6.74 (dd, *J* = 2.4 and 0.5 Hz, 1H, Ar*H*), 6.69 (dd, *J* = 8.4 and 2.4 Hz, 1H, Ar*H*), 3.89 (s, 3H, OCH_3), 3.83 (s, 3H, OCH_3), 3.79 (bs, 2H, NH_2).

^{1}H-NMR spectrum.

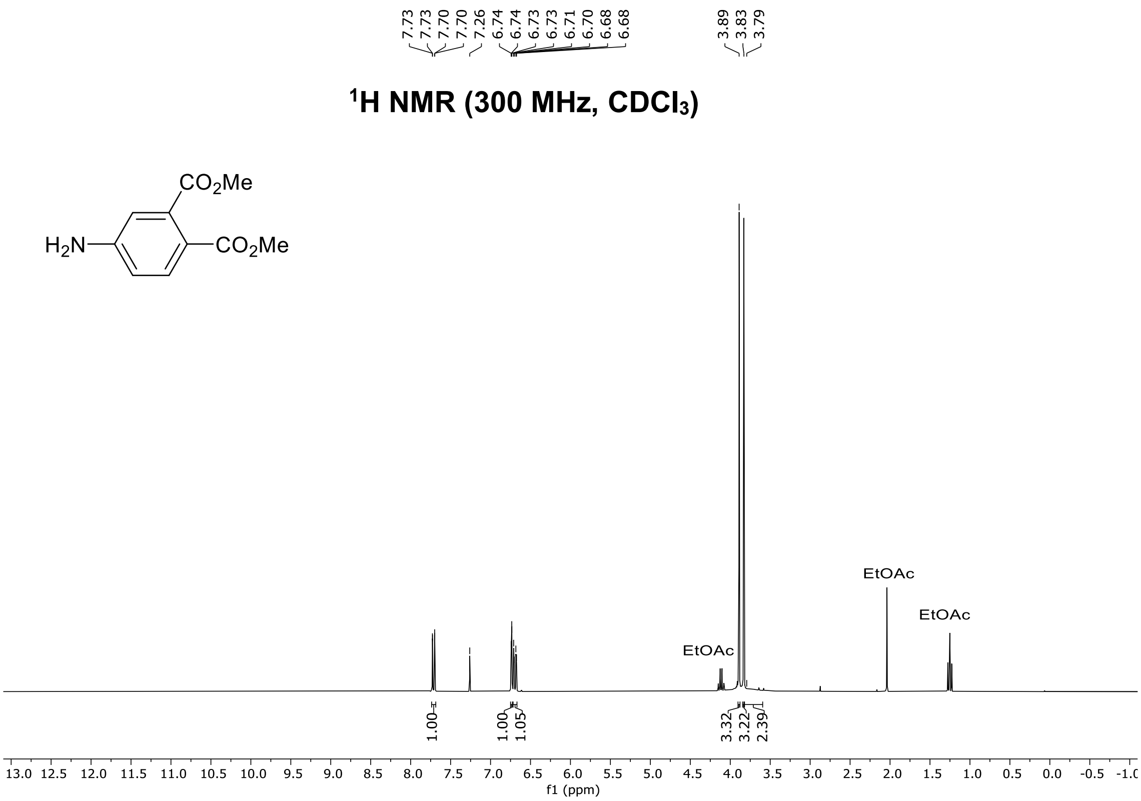

Dimethyl 3-aminophthalate:[5] Yielded (2.69 g, 33%) as a yellowish oil. After 12 h reaction time, the crude product was purified by silica gel flash chromatography (DCM/MeOH, 20/1).

^{1}H NMR (300 MHz, $CDCl_3$): δ = 7.24 (dd, J = 8.3 and 7.3 Hz, 1H, ArH), 6.90 (dd, J = 7.4 and 1.1 Hz, 1H, ArH), 6.78 (dd, J = 8.3 and 1.1 Hz, 1H, ArH), 5.19 (bs, 2H, NH_2), 3.86 (s, 3H, OCH_3), 3.84 (s, 3H, OCH_3).

^{1}H-NMR spectrum.

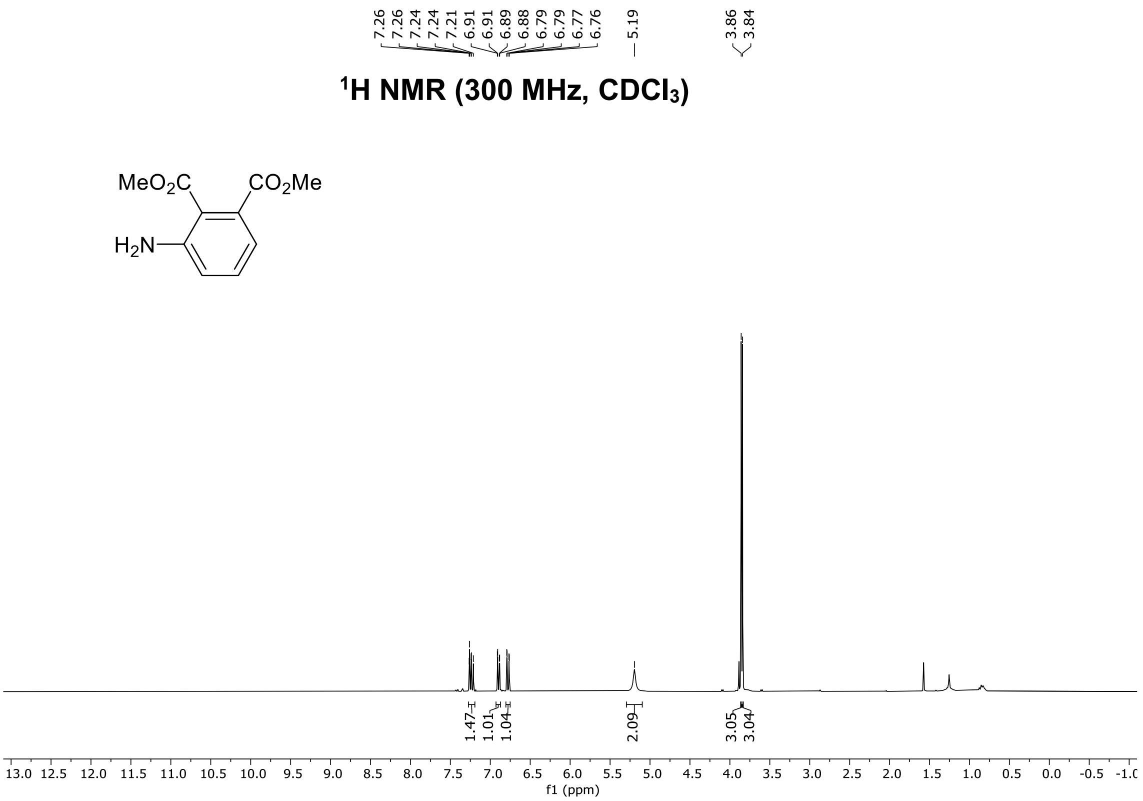

Synthesis and Characterization Data of Aryl Viologen Derivatives

*Synthesis of mono- and dicarboxyphenyl viologens **AV-3a-e**:*

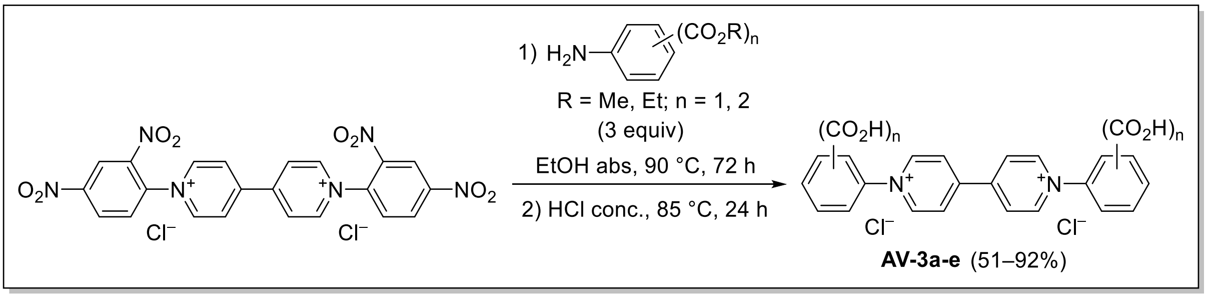

General procedure: In a round bottom flask (100 mL), 1,1′-bis(2,4-dinitrophenyl)-4,4′-bipyridinium dichloride (562 mg, 1 mmol), the appropriate alkyl aminobenzoate or dialkyl aminophthalate (3 equiv), and absolute EtOH (25 mL) were added under an inert N2 atmosphere. The reaction mixture was heated to 90 °C for 72 h. Then, the ethanol was removed by distillation. The residue was dissolved in water (25 mL) and washed three times with diethyl ether (20 mL). The aqueous phase was dried under vacuum. The resulting residue was mixed with concentrated HCl concentrated (10 mL) in a 25 mL round bottom flask. The mixture was then heated to 85 °C for 24 hours before cooling to room temperature. The

obtained solid product was isolated as the chloride salt by filtration and washed with acetone to provide the corresponding aryl viologen.

The spectral data of known compounds are in good agreement with the reported data.

1,1′-Bis(3-carboxyphenyl)-[4,4′-bipyridine]-1,1′-diium dichloride (AV-3a)[6]

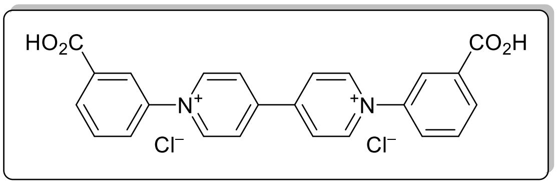

The general procedure was followed using methyl 3-aminobenzoate (454 mg, 3 mmol) yielded **AV-3a** (431 mg, 92%) as an orangish solid.

^{1}H NMR (300 MHz, D_2O): δ = 9.50 (d, *J* = 7.2 Hz, 4H, Ar*H*), 8.87 (d, *J* = 7.2 Hz, 4H, Ar*H*), 8.51−8.45 (m, 2H, Ar*H*), 8.42 (dt, *J* = 7.9 and 1.2 Hz, 2H, Ar*H*), 8.12 (ddd, *J* = 8.2, 2.5 and 1.1 Hz, 2H, Ar*H*), 7.93 (t, *J* = 8.1 Hz, 2H, Ar*H*).

<u>^{1}H-NMR spectrum</u>.

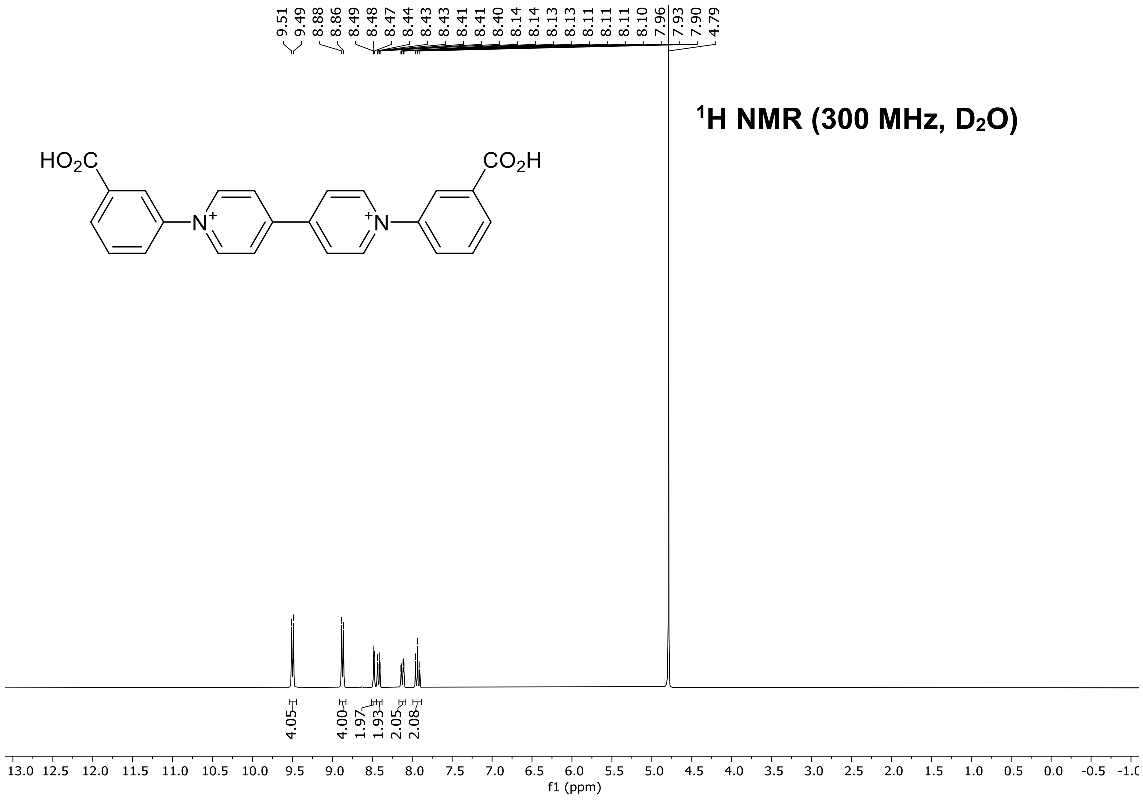

1,1′-Bis(4-carboxyphenyl)-[4,4′-bipyridine]-1,1′-diium dichloride (AV-3b)[7]

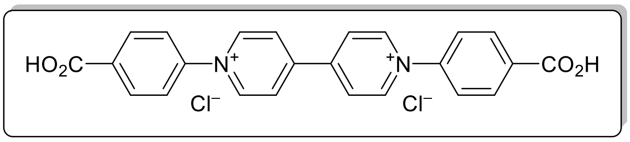

The general procedure was followed using ethyl 4-aminobenzoate (496 mg, 3 mmol) yielded **AV-3b** (380 mg, 81%) as a brownish solid.

^{1}H NMR (300 MHz, CD_3OD): δ = 9.64 (d, *J* = 7.2 Hz, 4H, Ar*H*), 8.98 (d, *J* = 7.2 Hz, 4H, Ar*H*), 8.43 (d, *J* = 8.9 Hz, 4H, Ar*H*), 8.07 (d, *J* = 8.9 Hz, 4H, Ar*H*).

^{1}H-NMR spectrum.

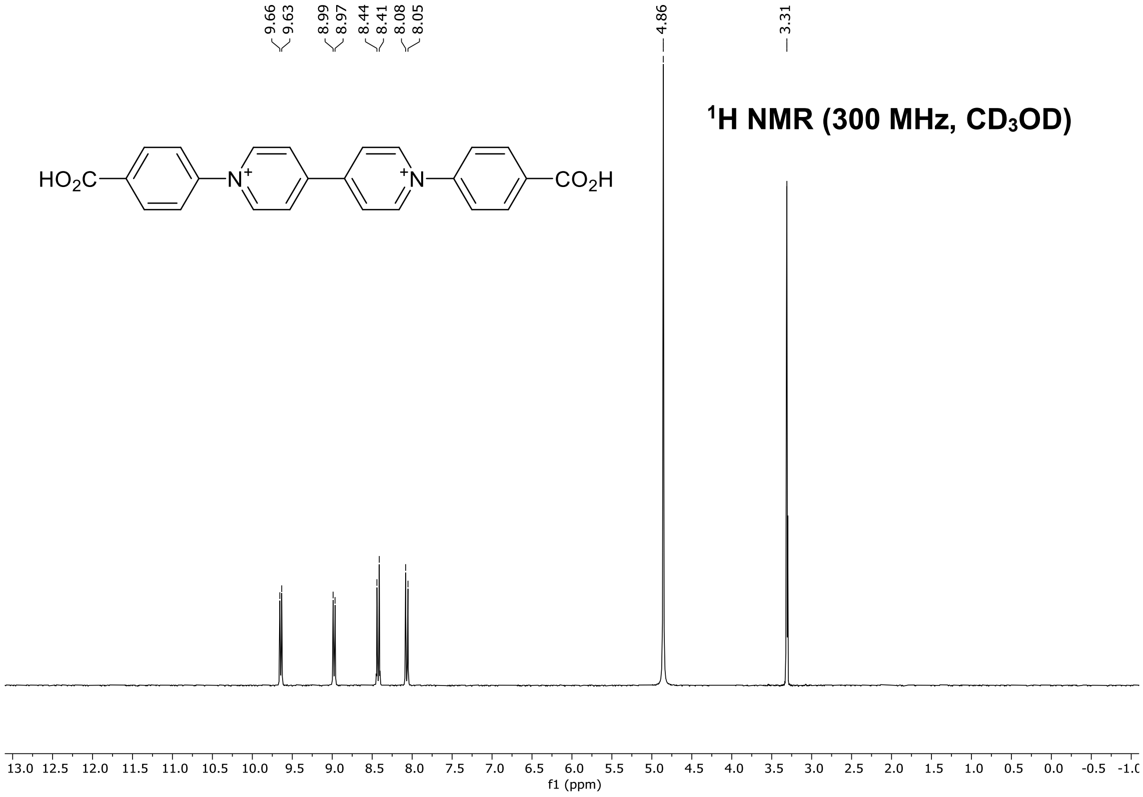

1,1′-Bis(3,5-dicarboxyphenyl)-[4,4′-bipyridine]-1,1′-diium dichloride (AV-3c)[8]

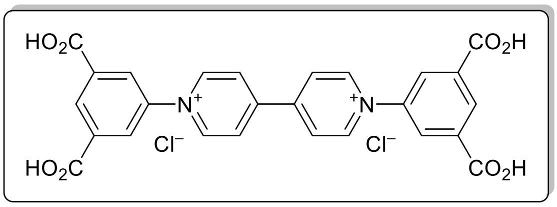

The general procedure was followed using dimethyl 5-aminoisophthalate (628 mg, 3 mmol) yielded **AV-3c** (472 mg, 85%) as a beige solid.

^{1}H NMR (300 MHz, DMSO-d_6): δ = 13.95 (bs, 4H, COO*H*), 9.81 (d, *J* = 6.4 Hz, 4H, Ar*H*), 9.15 (d, *J* = 6.4 Hz, 4H, Ar*H*), 8.76 (s, 2H, Ar*H*), 8.73 (s, 4H, Ar*H*).

^{1}H-NMR spectrum.

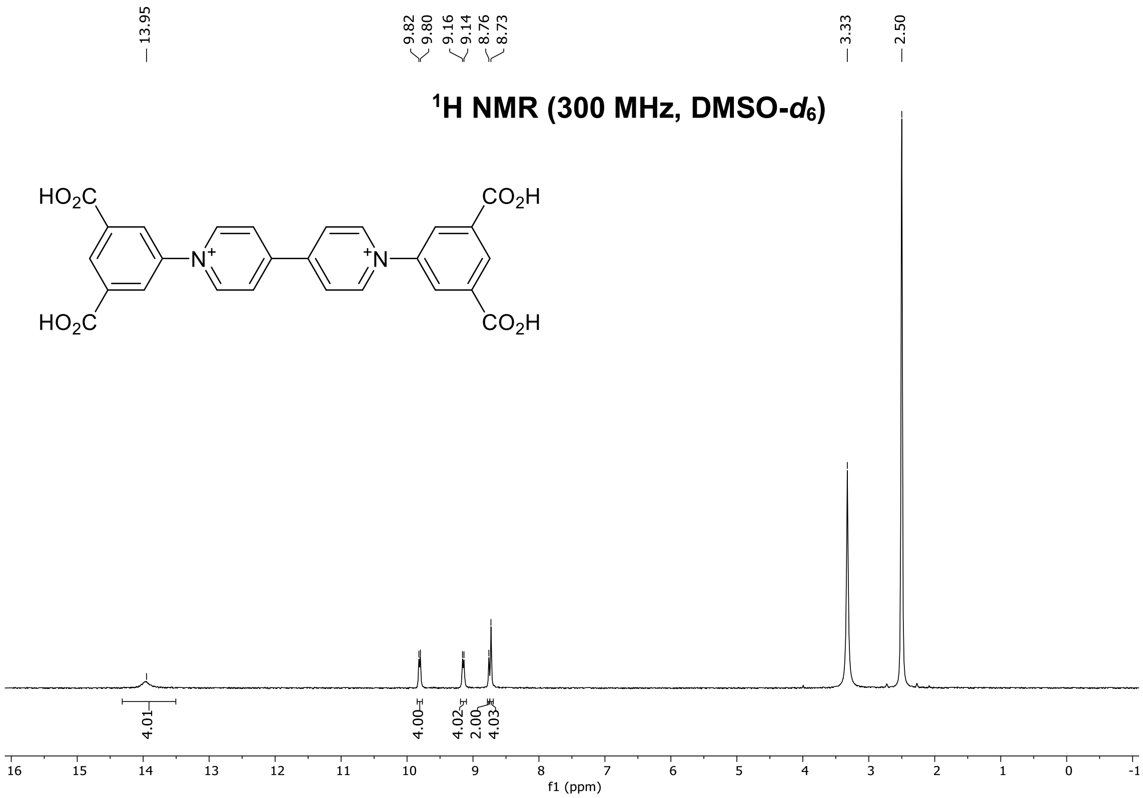

1,1′-Bis(3,4-dicarboxyphenyl)-[4,4′-bipyridine]-1,1′-diium dichloride (AV-3d)[9]

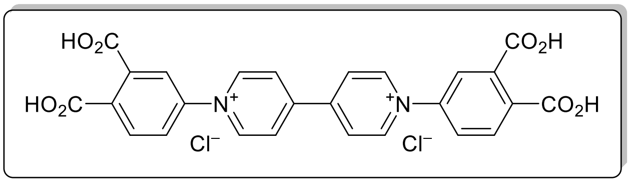

The general procedure was followed using dimethyl 4-aminophthalate (628 mg, 3 mmol) yielded **AV-3d** (477 mg, 86%) as a beige solid.

^{1}H NMR (300 MHz, DMSO-d_6): δ = 9.78 (d, J = 6.8 Hz, 4H, ArH), 9.15 (d, J = 6.9 Hz, 4H, ArH), 8.35 (d, J = 2.3 Hz, 2H, ArH), 8.20 (dd, J = 8.3 and 2.4 Hz, 2H, ArH), 8.07 (d, J = 8.3 Hz, 2H, ArH).

^{1}H-NMR spectrum.

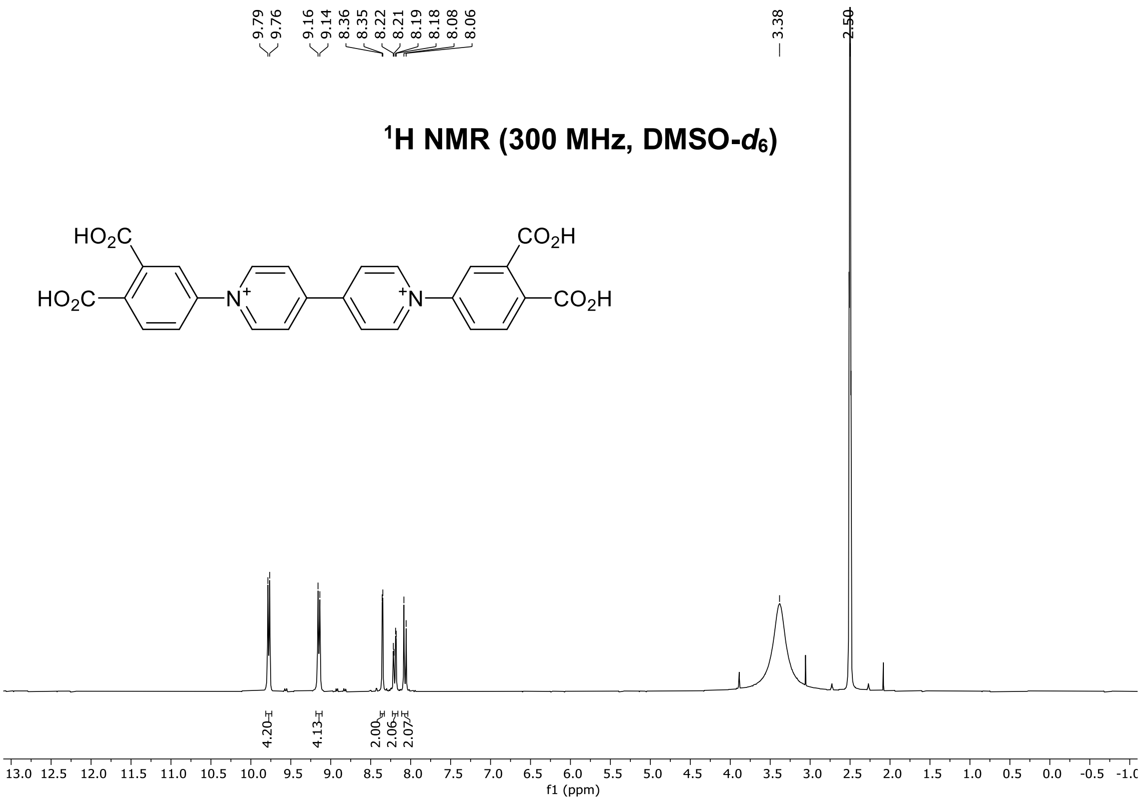

1,1′-Bis(2,3-dicarboxyphenyl)-[4,4′-bipyridine]-1,1′-diium dichloride (AV-3e)

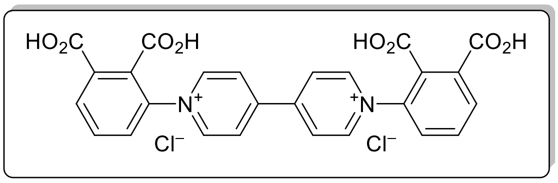

The general procedure was followed using dimethyl 3-aminophthalate (628 mg, 3 mmol) yielded **AV-3e** (430 mg, 77%) as an off-white solid, Mp (from H_2O) 306–308 ºC (decomp.).

^{1}H NMR (300 MHz, DMSO-d_6): δ =9.61 (d, *J* = 6.1 Hz, 4H, Ar*H*), 9.01 (d, *J* = 6.1 Hz, 4H, Ar*H*), 8.32 (d, *J* = 8.3 Hz, 2H, Ar*H*), 7.98 (d, *J* = 8.3 Hz, 2H, Ar*H*), 7.82 (d, *J* = 8.3 Hz, 2H, Ar*H*)

^{13}C NMR (75.4 MHz, CD_3OD): δ =167.6 (2 × C), 166.0 (2 × C), 152.9 (2 × C), 149.1 (4 × CH), 147.4 (2 × C), 141.1 (2 × C), 137.6 (2 × C), 134.4 (2 × CH), 133.9 (2 × CH), 132.8 (2 × CH), 128.2 (4 × CH).

^{1}H-NMR spectrum.

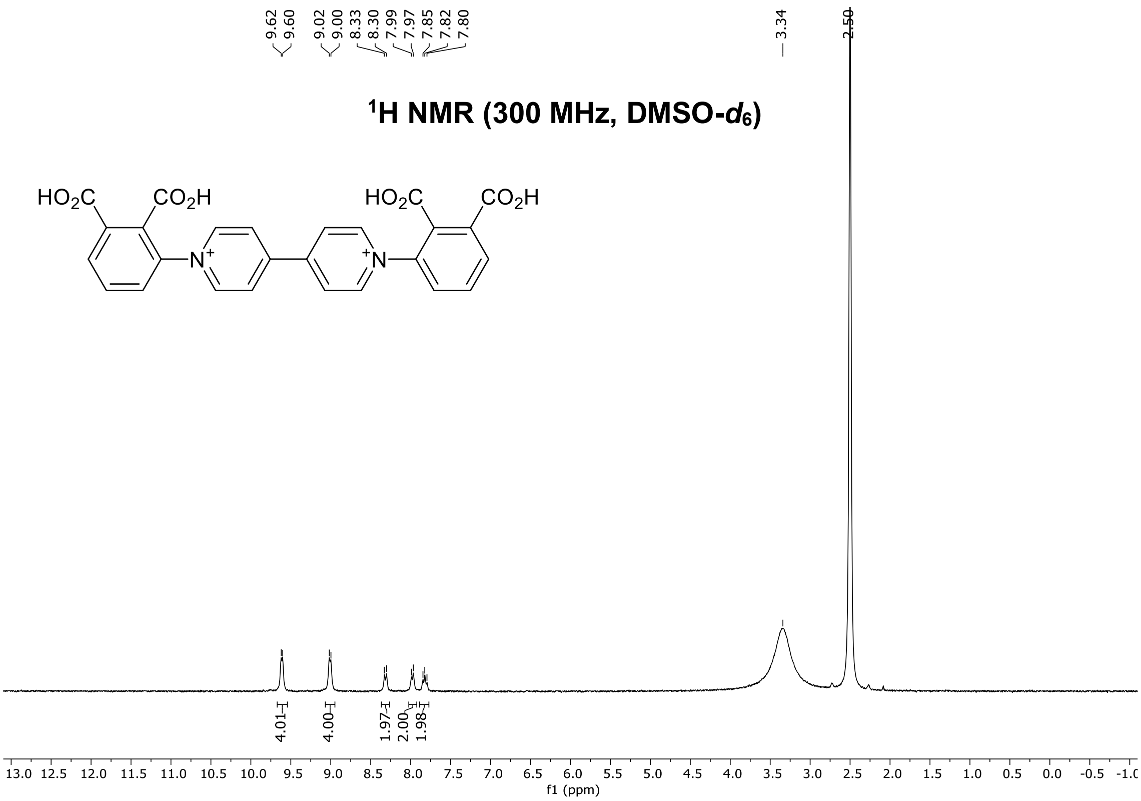

^{13}C-NMR spectrum.

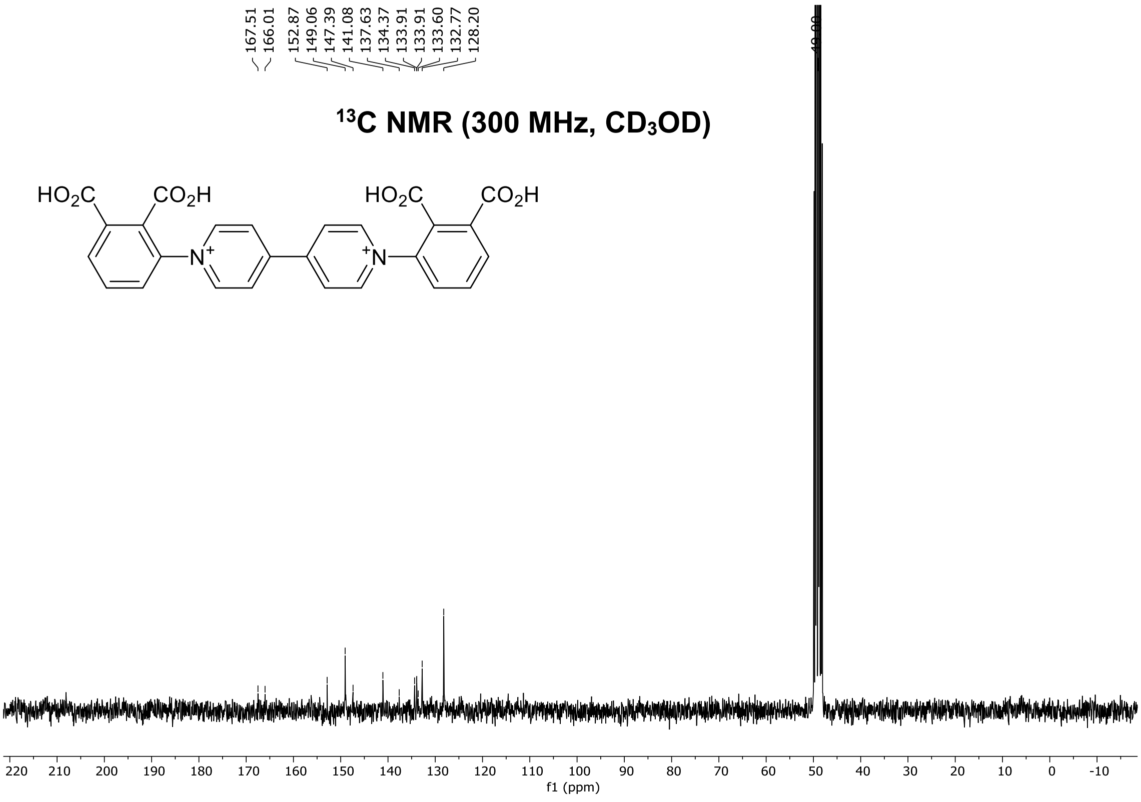

*Synthesis and Characterization Data of Phenyl Viologen **PV**:*

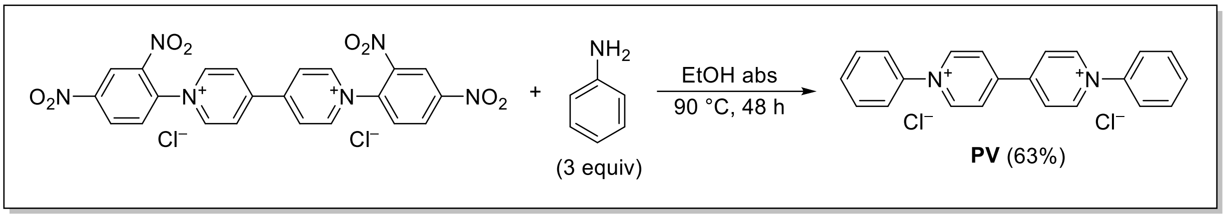

In a round bottom flask (100 mL), 1,1′-bis(2,4-dinitrophenyl)-4,4′-bipyridinium dichloride (562 mg, 1 mmol), absolute EtOH (25 mL) and aniline (280 mg, 3 mmol) were added under an inert N_2 atmosphere. The reaction mixture was heated to 90 °C for 48 h. Then, the ethanol was removed by distillation. The residue was dissolved in water (25 mL) and washed three times with diethyl ether (20 mL). The aqueous phase was dried under vacuum and the crude product **PV** was purified by recrystallization form H_2O.

The spectral data of the compound are in good agreement with the reported data.[10]

1,1′-Diphenyl-[4,4′-bipyridine]-1,1′-diium dichloride: Yielded (240 mg, 63%) as a reddish solid.

^{1}H NMR (300 MHz, D_2O): δ = 9.45 (d, *J* = 7.2 Hz, 4H, Ar*H*), 8.84 (d, *J* = 7.2 Hz, 4H, Ar*H*), 7.91−7.78 (m, 10H, Ar*H*).

^{1}H-NMR spectrum.

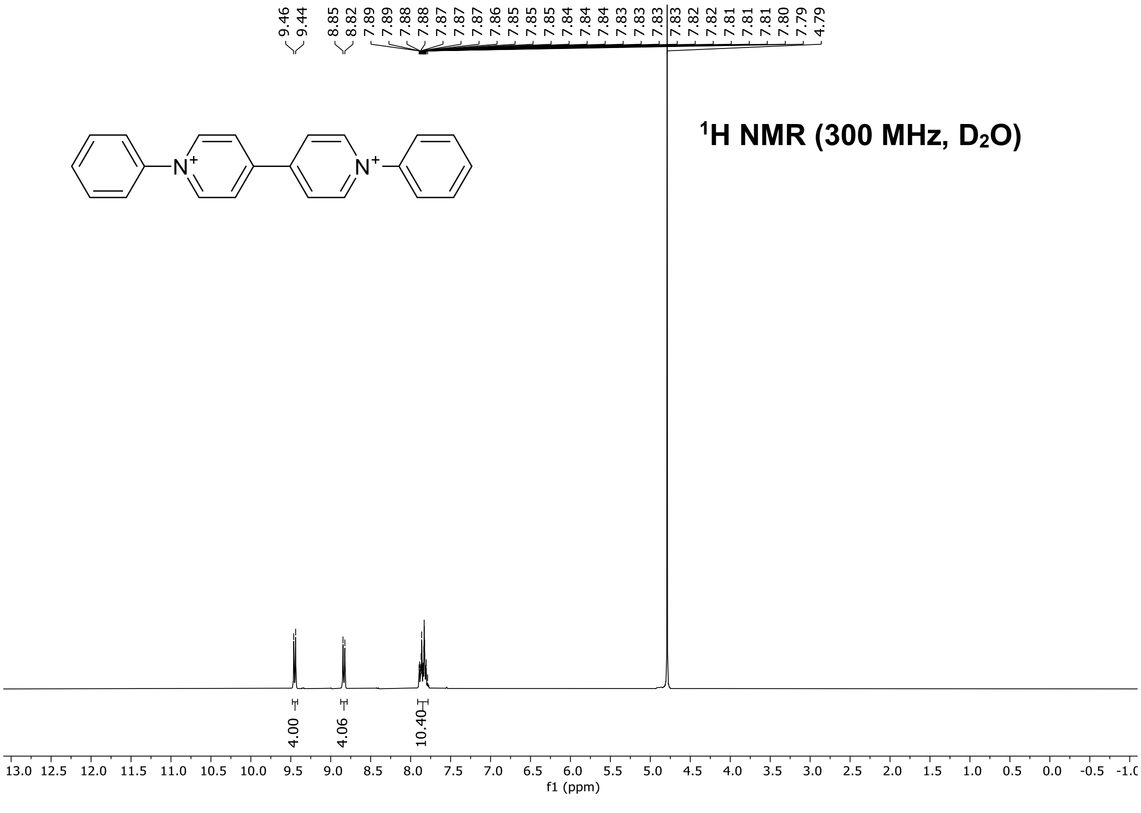

Synthesis and Characterization Data of 2,5-Dihydroxyphenyl Viologen **B-2,5-DHPV** ***(AV-3f)*****:**

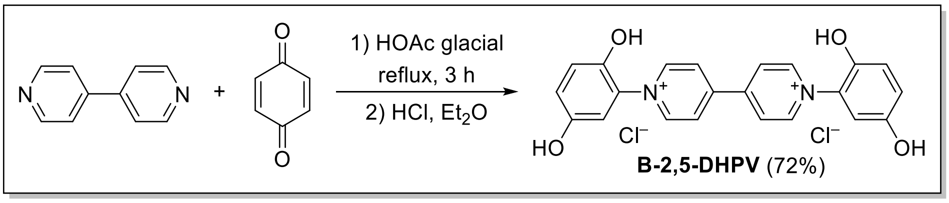

2,5-Dihydroxyphenyl viologen (**B-2,5-DHPV**) was prepared according to a modified procedure of a reported method.[ref. 33 main text]

In a round bottom flask (250 mL), 4,4′-bipyridine (1 g, 6.4 mmol) was added to a stirred mixture of *p*-benzoquinone (1.38 g, 12.8 mmol) and glacial acetic acid (25 mL). The reaction mixture was heated under reflux for 3 h and after this time excess conc. HCl was added. After cooling to room temperature, diethyl ether (150 mL) was added to the mixture and the obtained deep purple solid was filtered off, washed with cold water and dried under vacuum. Purification by recrystallization from H_2O/acetone gave rise to the desired product **B-2,5-DHPV** (**AV-3f**).

The spectral data of the compound are in good agreement with the reported data.[ref. 33 main text]

1,1′-Bis(2,5-dihydroxyphenyl)-[4,4′-bipyridine]-1,1′-diium dichloride: Yielded (2 g, 72%) as a deep purple solid.

^{1}H NMR (300 MHz, DMSO-*d*$_6$): δ = 10.48 (s, 2H, O*H*), 9.71 (s, 2H, O*H*), 9.52 (d, *J* = 6.7 Hz, 4H, Ar*H*), 8.95 (d, *J* = 6.7 Hz, 4H, Ar*H*), 7.13 (d, *J* = 2.9 Hz, 2H, Ar*H*), 7.11 (d, *J* = 9.0 Hz, 2H, Ar*H*), 7.01 (dd, *J* = 8.9 and 2.8 Hz, 2H, Ar*H*).

^{13}C NMR (75.4 MHz, DMSO-*d*$_6$): δ =150.4 (2 × C), 149.4 (2 × C), 147.4 (4 × CH), 142.6 (2 × C), 129.6 (2 × C), 126.6 (4 × CH), 119.8 (2 × CH), 118.2 (2 × CH), 112.7 (2 × CH).

^{1}H-NMR spectrum.

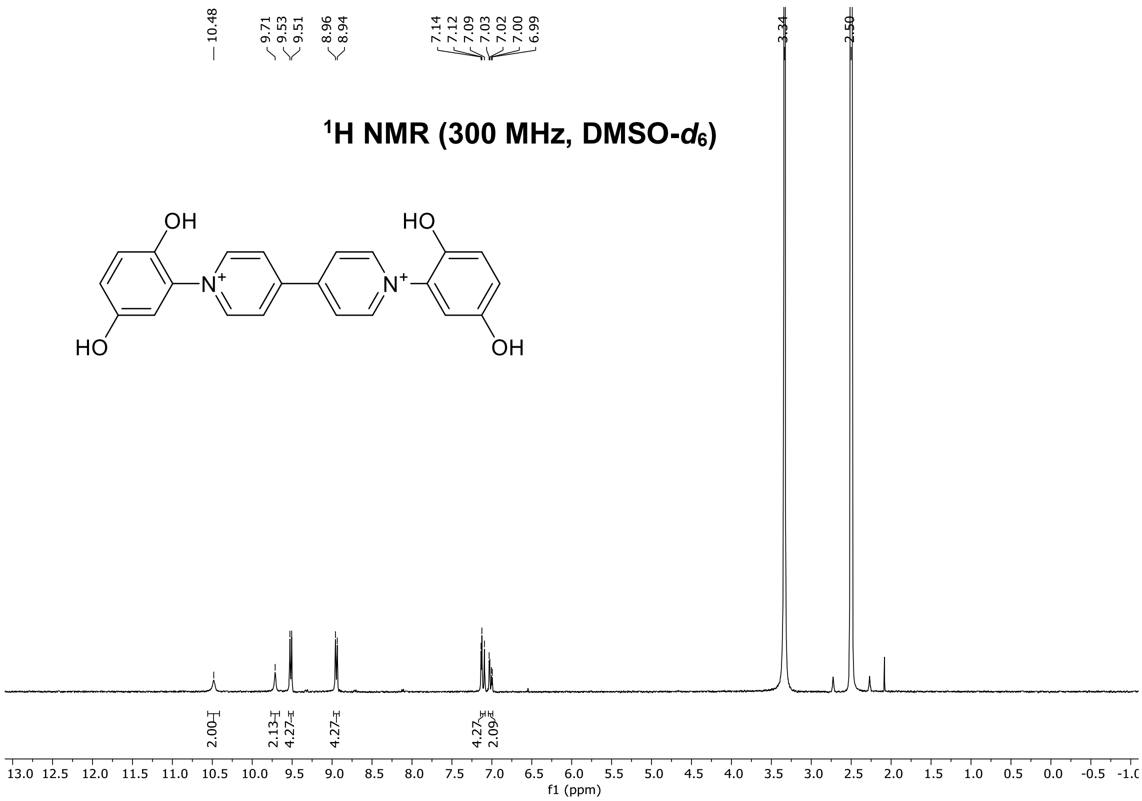

^{13}C-NMR spectrum.

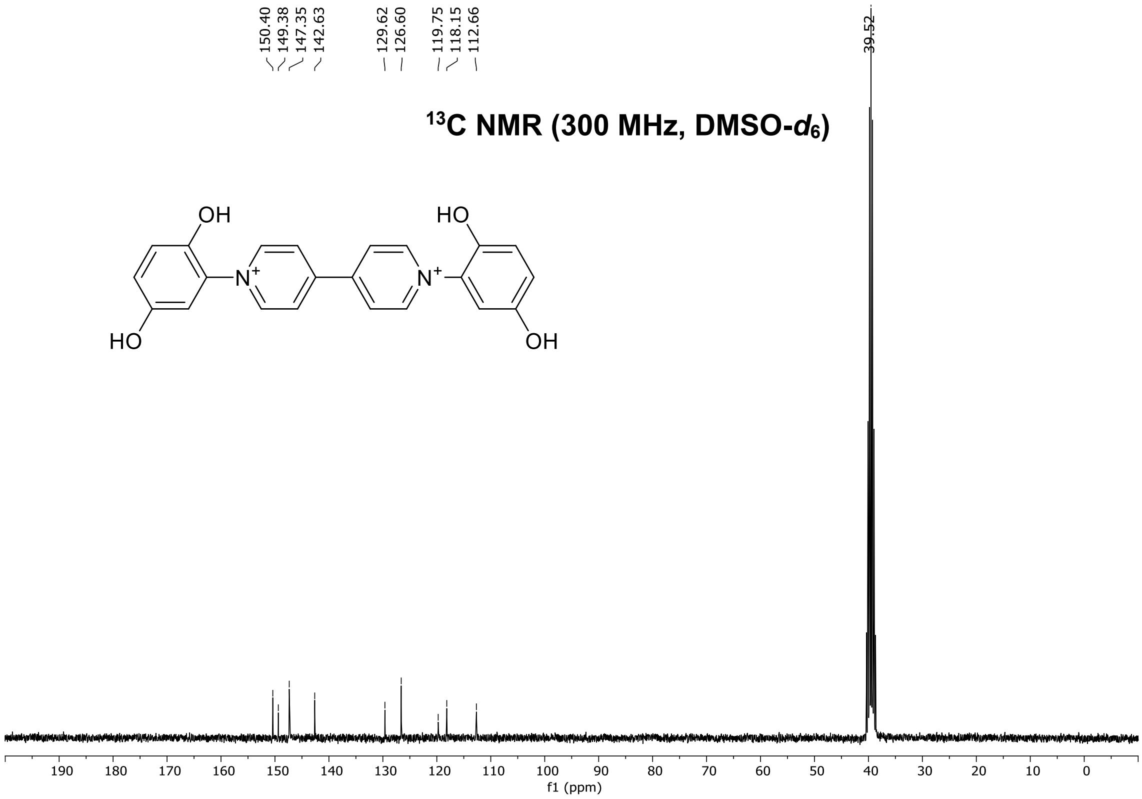

Synthesis and Characterization Data of Viologen $(SPr)_2V$:

Synthesis.

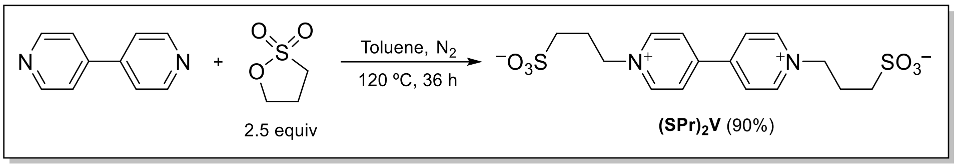

1,1′-Bis(3-sulfonatopropyl) viologen (**$(SPr)_2V$**) was prepared according to a reported method.[ref. 27 main text]

In a round bottom flask (250 mL), 1,3-propanesultone (9.8 g, 80 mmol) was dissolved in anhydrous toluene (60 mL) under an inert nitrogen atmosphere and the obtained solution was heated at 110 ºC. Then, a solution of 4,4′-bipyridine (5 g, 32 mmol) in anhydrous toluene (40 mL) was added slowly with stirring. The reaction mixture was heated at reflux (oil bath, 120 ºC) with vigorous stirring for 36 h. Upon cooling to room temperature, the resulting white precipitate was filtered off, washed several times with hot MeOH and dried under vacuum.

The spectral data of the compound are in good agreement with the reported data.[ref. 27 main text]

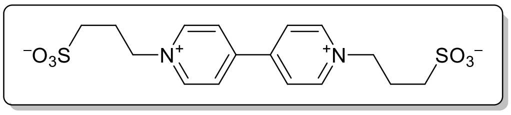

1,1′-Bis(3-sulfonatopropyl)-4,4′-bipyridinium: Yielded (11.22 g, 90%) as a white solid.

^{1}H NMR (300 MHz, D_2O): δ = 9.18 (d, *J* = 6.9 Hz, 4H, Ar*H*), 8.59 (d, *J* = 6.6 Hz, 4H, Ar*H*), 4.92 (t, *J* = 7.4 Hz, 4H, CH_2), 3.06 (t, *J* = 7.2 Hz, 4H, CH_2), 2.56 (ap, *J* = 7.3 Hz, 4H, CH_2).

^{1}H-NMR spectrum.

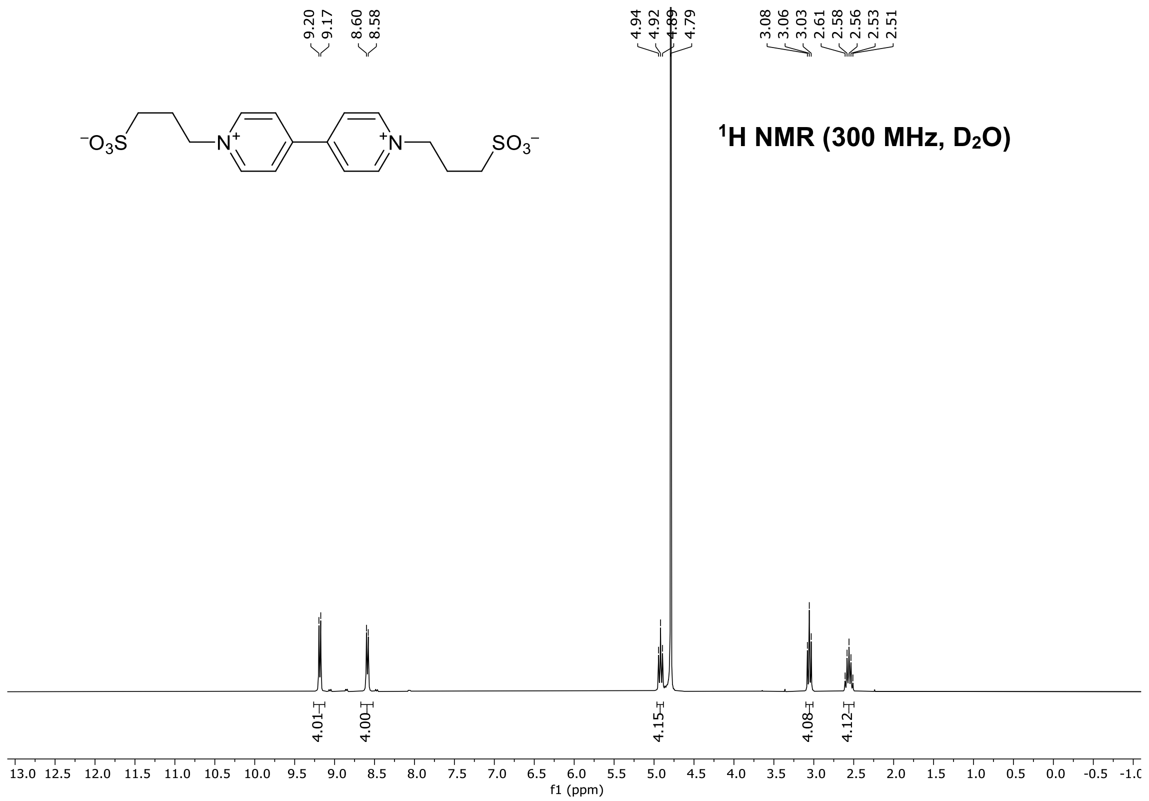

Synthesis and Characterization Data of 1,3-Butanesultone:

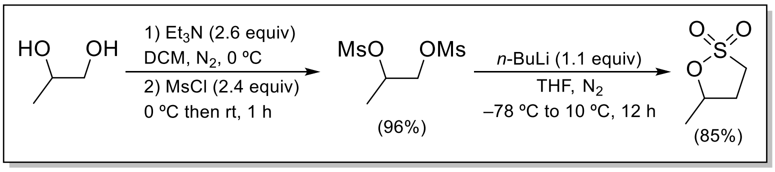

1,3-Butanesultone was prepared in two steps from propylene glycol according to a reported method.[ref. 27 main text]

Synthesis.

Procedure for the synthesis of propane-1,2-diyl dimethanesulfonate: In an oven dried Schlenck flask (250 mL), propylene glycol (4.56 g, 60 mmol) and anhydrous DCM (150 mL) were added under an inert nitrogen atmosphere. The resulting solution was cooled to 0 ºC and Et_3N (21.9 mL, 156 mmol) was added. Then, methanesulfonyl chloride (MsCl; 11.1 mL, 144 mmol) was added slowly via a syringe under vigorous stirring and the mixture was stirred at 0 ºC for 1 h. The obtained suspension was allowed to warm and stirred at room temperature for 1 h. The reaction was quenched with water (100 mL). The layers were separated and the aqueous layer was extracted with DCM (2 × 75 mL). The combined organic layers were washed with water and brine, dried over anhydrous Na_2SO_4, filtered and concentrated under

reduced pressure. The product was obtained pure and used in the next step without need for further purification. The product is purified by crystallization (toluene/hexane), if required.

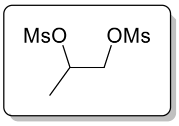

Propane-1,2-diyl dimethanesulfonate: Yielded (13.38 g, 96%) as a yellowish solid.

^{1}H NMR (300 MHz, $CDCl_3$): δ = 4.99 (pd, *J* = 6.6 and 3.1 Hz, 1H, C*H*), 4.32 (dd, *J* = 11.6 and 3.1 Hz, 1H, CH*H*), 4.22 (dd, *J* = 11.6 and 6.7 Hz, 1H, C*H*H), 3.07 (s, 3H, CH_3), 3.06 (s, 3H, CH_3), 1.46 (d, *J* = 6.6 Hz, 1H, CH_3).

^{13}C NMR (75.4 MHz, $CDCl_3$): δ = 75.8 (CH), 70.5 (CH_2), 38.8 (CH_3), 37.9 (CH_3), 17.5 (CH_3).

LR-MS (EI), *m/z* (%): 158 (25), 123 (100), 79 (88).

<u>*^{1}H-NMR spectrum*</u>.

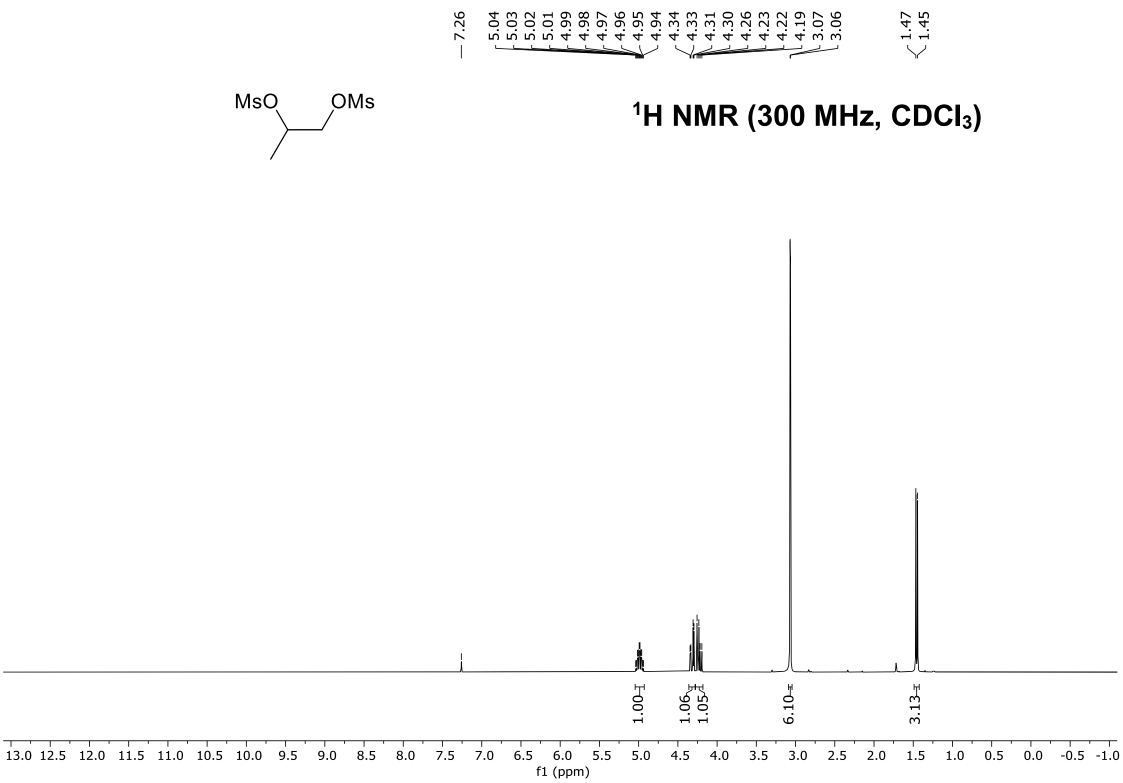

<u>*^{13}C-NMR spectrum*</u>.

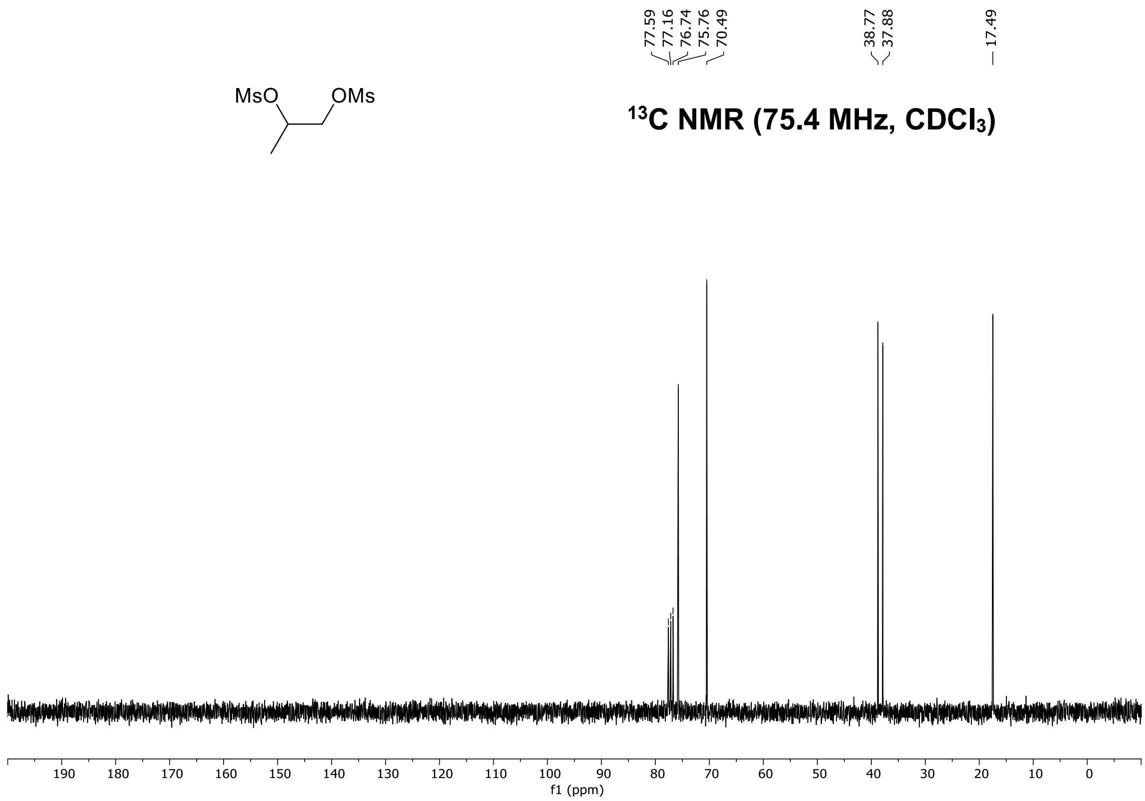

Procedure for the synthesis of 1,3-Butanesultone: In an oven dried Schlenck flask (250 mL), propane-1,2-diyl dimethanesulfonate (11.6 g, 50 mmol) and anhydrous THF (150 mL) were added under an inert nitrogen atmosphere. The resulting solution was cooled to −78 ºC and *n*-BuLi (22 mL of a 2.5 M solution in hexanes, 55 mmol) was added under vigorous stirring. Then, the obtained suspension was stirred at 10 ºC for 12 h. The reaction was quenched with water (100 mL) and the reaction mixture was extracted with DCM (3 × 70 mL). The combined organic layers were washed with brine, dried over anhydrous Na_2SO_4, filtered and concentrated under reduced pressure giving rise to the desired product. The sultone was obtained in pure form and used without further purification in the next step. Alternatively, the addition of *n*-BuLi can be carried out at higher temperature, although a lower yield was obtained and the crude product should be further purified.

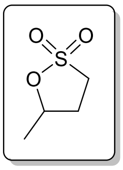

1,3-Butanesultone: Yielded (5.8 g, 85%) as a yellowish oil.

^{1}H NMR (300 MHz, $CDCl_3$): δ = 4.78 (dq, *J* = 8.7 and 6.1 Hz, 1H, C*H*), 3.40–3.32 (m, 1H, CH*H*), 3.27 (ddd, *J* = 13.1, 9.3 and 7.9 Hz, 1H, C*H*H), 2.63 (dddd, *J* = 13.5, 7.9, 5.9 and 4.4 Hz, 1H, CH*H*), 2.28 (dq, *J* = 13.1 and 9.0 Hz, 1H, C*H*H), 1.53 (d, *J* = 6.1 Hz, 3H, C*H*$_3$).

^{13}C NMR (75.4 MHz, $CDCl_3$): δ = 79.2 (CH), 46.1 (CH_2), 31.3 (CH_2), 20.9 (CH_3).

LR-MS (EI), *m/z* (%): 121 (100), 43 (81), 29 (42).

<u>*^{1}H-NMR spectrum*</u>.

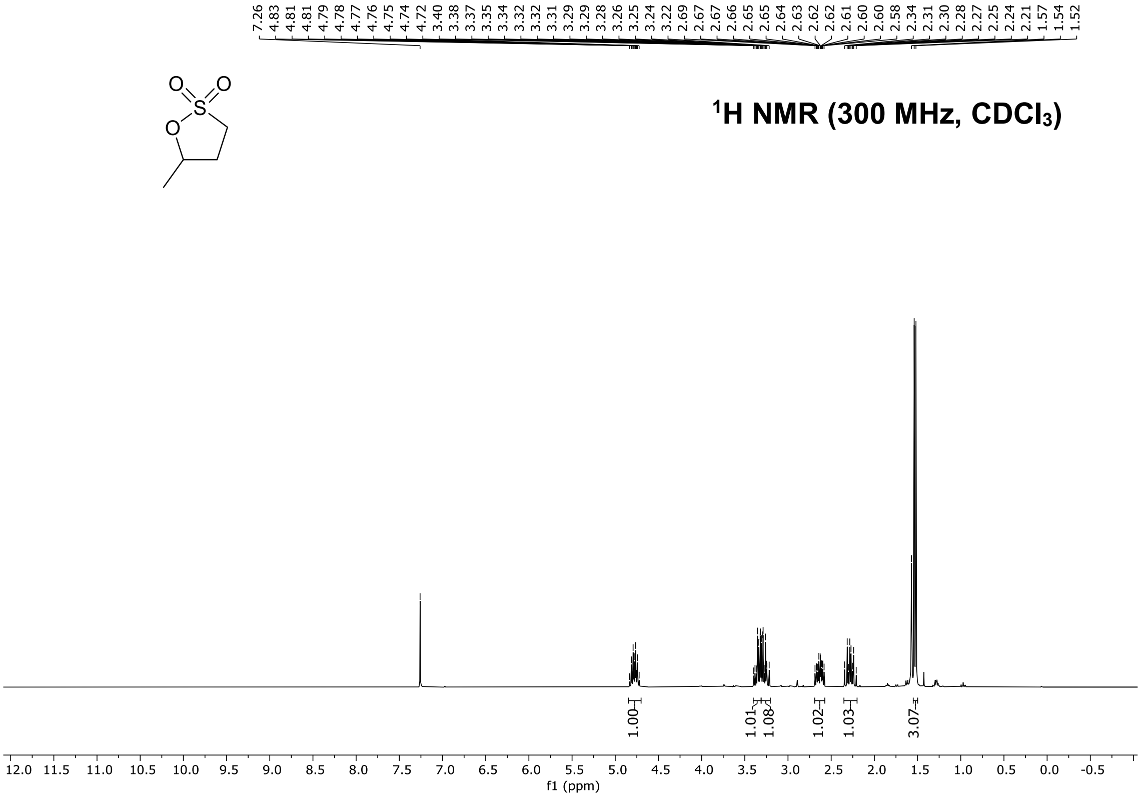

^{13}C-NMR spectrum.

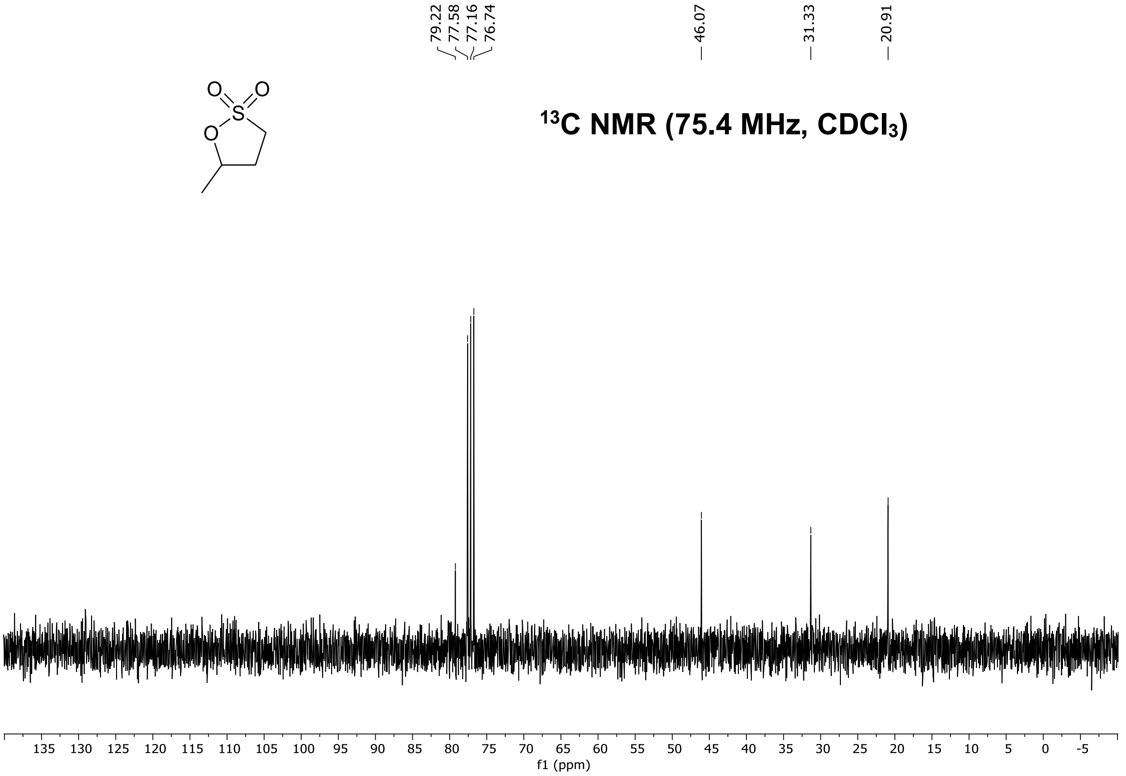

Synthesis and Characterization Data of Viologen BS3Bu-Vi:

Synthesis.

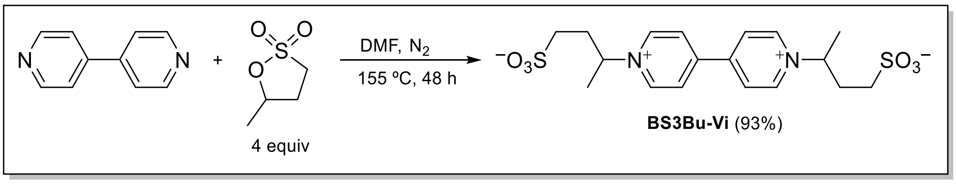

1,1′-Bis-3-sulfonatobutyl viologen (**BS3Bu-Vi**) was prepared according to a reported method.[ref. 27 main text]

In a screw-cap Schlenk flask (48 mL), 1,3-butanesultone (2.18 g, 16 mmol) was dissolved in anhydrous DMF (25 mL) under an inert nitrogen atmosphere. Then, 4,4’-bipyridine (625 mg, 4 mmol) was added with stirring to the resulting solution. The Schlenk flask was sealed and the reaction mixture was heated at 155 ºC with vigorous stirring for 48 h. Upon cooling to room temperature, the resulting white precipitate was filtered off, washed several times with MeOH and dried under vacuum.

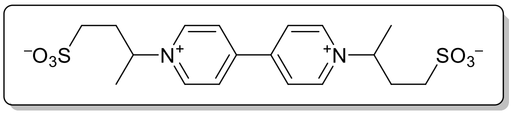

1,1'-Bis(1-methyl-3-sulfonatopropyl)-4,4'-bipyridinium: Yielded (1.59 g, 93%) as a white solid, Mp (from MeOH) 296–298 ºC (decomp.).

^{1}H NMR (300 MHz, D_2O): δ = 9.23 (d, *J* = 6.7 Hz, 4H, Ar*H*), 8.61 (d, *J* = 6.7 Hz, 4H, Ar*H*), 5.20 (h, *J* = 7.0 Hz, 2H, C*H*), 3.02–2.81 (m, 4H, C*H₂*), 2.55 (q, *J* = 7.3 Hz, 4H, C*H₂*), 1.82 (d, *J* = 6.7 Hz, 6H, C*H₃*).

^{13}C NMR (75.4 MHz, D_2O): δ = 151.1 (2 × C), 144.8 (4 × CH), 128.0 (4 × CH), 68.7 (2 × CH), 47.4 (2 × CH_2), 32.4 (2 × CH_2), 21.0 (2 × CH_3).

HR-MS (ESI+) *m/z*: $[M+H]^+$ calcd. for $C_{18}H_{25}N_2O_6S_2$, 429.1149; found, 429.1153.

^{1}H-NMR spectrum.

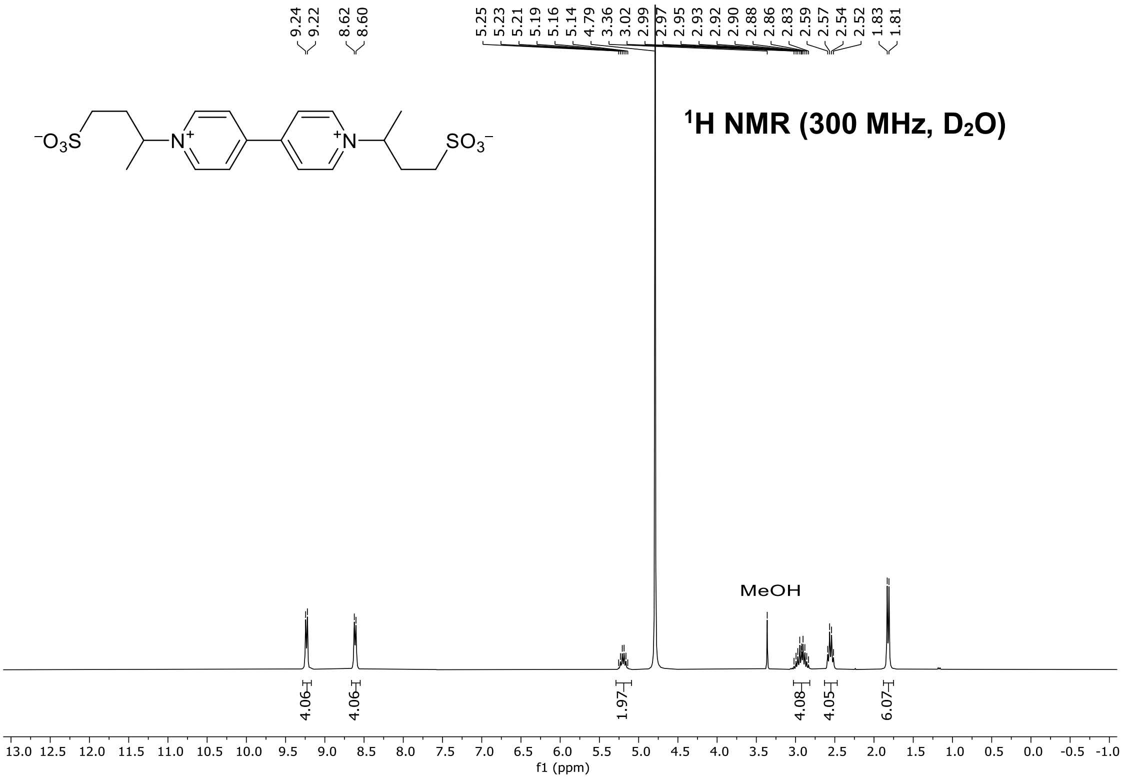

^{13}C-NMR spectrum.

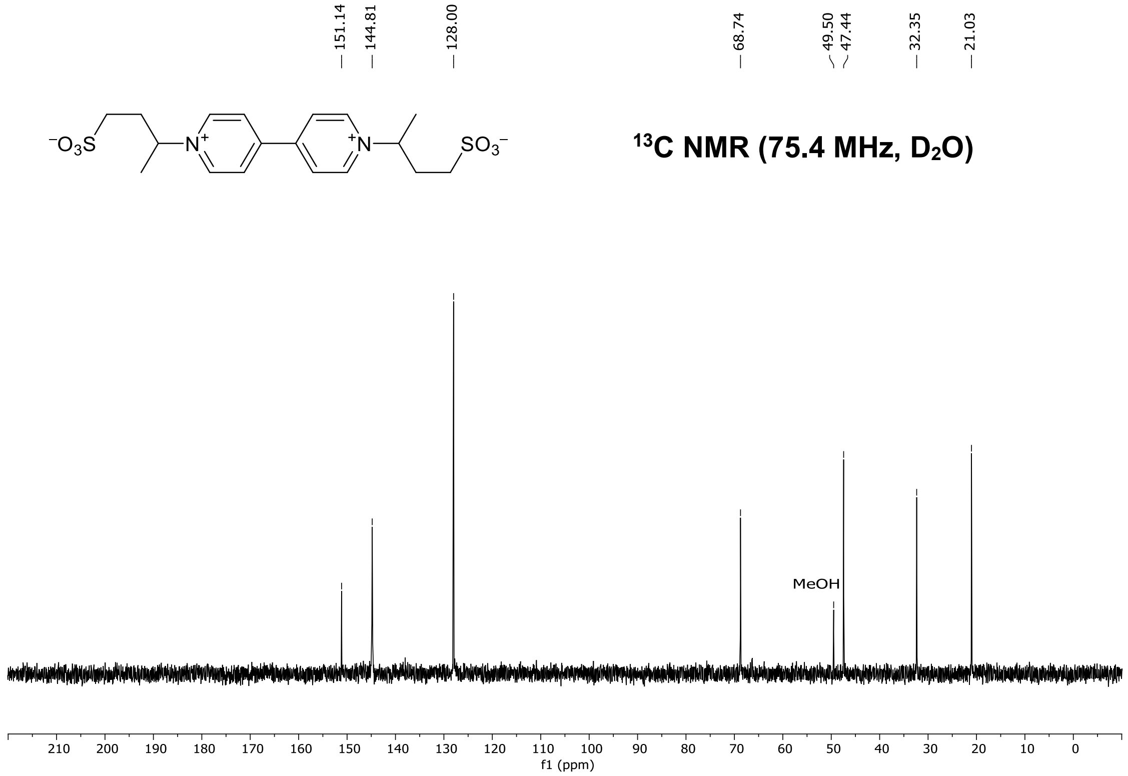

Synthesis and Characterization Data of 4-Amino-1,1-dimethylpiperidin-1-ium iodide:

Synthesis.

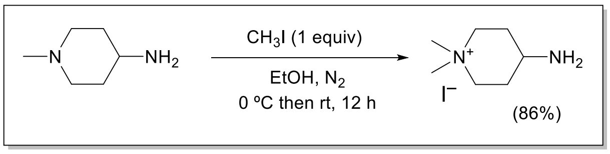

4-Amino-1,1-dimethylpiperidin-1-ium iodide was prepared from propylene glycol according to a modified procedure of a reported method.[11]

In an oven dried Schlenck flask (50 mL), 1-methylpiperidin-4-amine (1.85 g, 16.3 mmol) and absolute EtOH (11 mL) were added under an inert nitrogen atmosphere. The resulting solution was cooled to 0 ºC and CH_3I (1 mL, 16.1 mmol) was added slowly under vigorous stirring. The obtained suspension was allowed to warm and stirred at room temperature for 24 h. The resulting whitish precipitate was filtered off, washed several times with EtOH and dried under vacuum.

The spectral data of the compound are in good agreement with the reported data.[11]

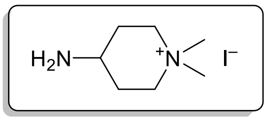

4-Amino-1,1-dimethylpiperidin-1-ium iodide: Yielded (3.55 g, 86%) as a whitish solid.

^{1}H NMR (300 MHz, D_2O): δ = 3.63–3.53 (m, 2H, NCH*H*), 3.45 (td, *J* = 13.1 and 3.4 Hz, 2H, NC*H*H), 3.37–3.26 (m, 1H, C*H*), 3.20 (s, 3H, C*H$_3$*), 3.15 (s, 3H, C*H$_3$*), 2.26–2.09 (m, 2H, CH*H*), 1.99 (dq, *J* = 11.7 and 4.1 Hz, 2H, C*H*H).

^{13}C NMR (75.4 MHz, D_2O): δ = 60.9 (2 × CH_2), 55.5 (CH_3), 47.7 (CH_3), 44.6 (CH), 26.1 (2 × CH_2).

<u>*^{1}H-NMR spectrum*</u>.

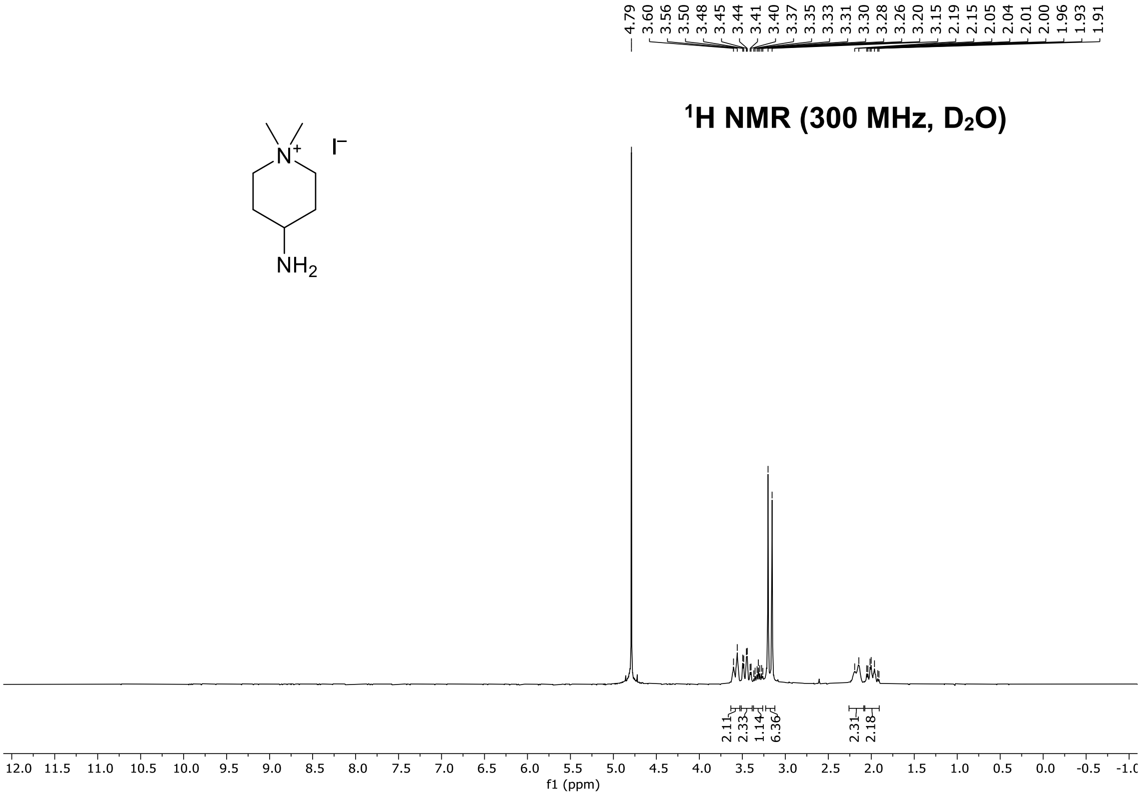

^{13}C-NMR spectrum.

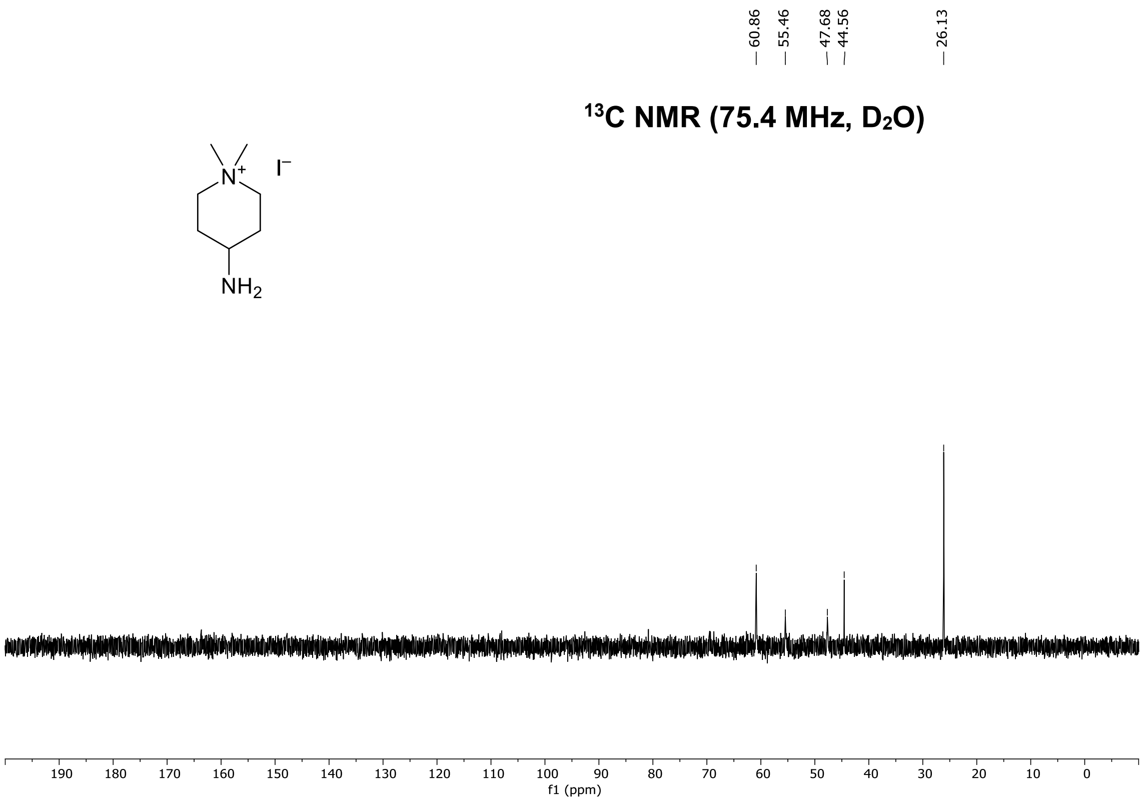

Synthesis and Characterization Data of Viologen (DBPPy)Cl$_4$:

Synthesis.

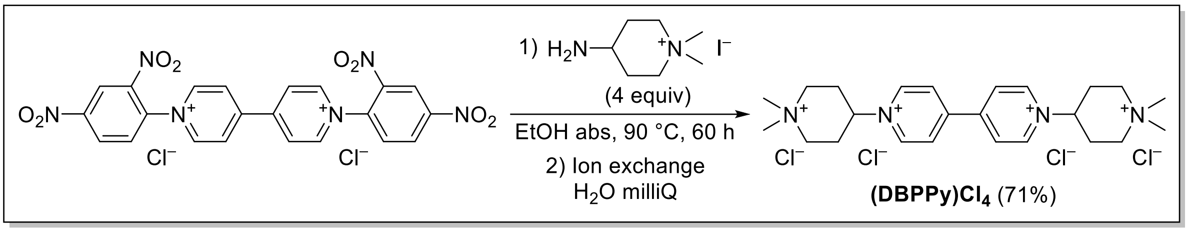

1,1′-Bis(1,1-dimethylpiperidin-1-ium-4-yl) viologen (**(DBPPy)Cl$_4$**) was prepared according to a modified procedure of a reported method.[ref. 36 main text]

In a round bottom flask (50 mL), 1,1′-bis(2,4-dinitrophenyl)-4,4′-bipyridinium dichloride (281 mg, 0.5 mmol), 4-amino-1,1-dimethylpiperidin-1-ium iodide (512 mg, 2 mmol), and absolute EtOH (15 mL) were added under an inert N_2 atmosphere. The reaction mixture was heated to 90 ºC for 60 h. Then, the EtOH was removed by distillation. The residue was dissolved in water (10 mL) and washed three times with diethyl ether (15 mL). The aqueous phase was poured over excess MeOH and the resulting red precipitate was filtered off, washed several times with MeOH and dried under vacuum. The red solid obtained was dissolved in H_2O Milli-Q (3 mL)

and submitted to an ion-exchange chromatography with wet Amberlite IRA-900(Cl) (12 g) and H_2O Milli-Q (25 mL) as eluent. Then, the water was evaporated under reduced pressure and the desired product was dried under vacuum.

The spectral data of the compound are in good agreement with the reported data.[ref. 36 main text]

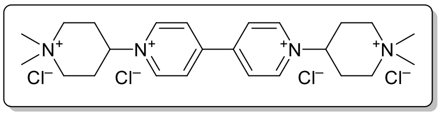

1,1'-Bis(1,1-dimethylpiperidin-1-ium-4-yl)-4,4'-bipyridinium tetrachloride: Yielded (186 mg, 71%) as a yellowish solid.

^{1}H NMR (300 MHz, D_2O): δ = 9.36 (d, *J* = 7.1 Hz, 4H, Ar*H*), 8.70 (d, *J* = 7.0 Hz, 4H, Ar*H*), 5.29 (tt, *J* = 12.6 and 4.3 Hz, 2H, C*H*), 3.94–3.72 (m, 8H, NC*HH*), 3.38 (d, *J* = 10.3 Hz, 12H, C*H_3*), 2.85 (dq, *J* = 13.5 and 4.4 Hz, 4H, CH*H*), 2.75–2.60 (m, 4H, C*H*H*)*.

^{13}C NMR (75.4 MHz, D_2O): δ = 150.8 (2 × C), 144.5 (4 × CH), 127.7 (4 × CH), 65.9 (2 × CH), 61.1 (4 × NCH_2), 56.4 (2 × CH_3), 47.7 (2 × CH_3), 26.5 (2 × CH_2).

<u>^{1}H-NMR spectrum</u>.

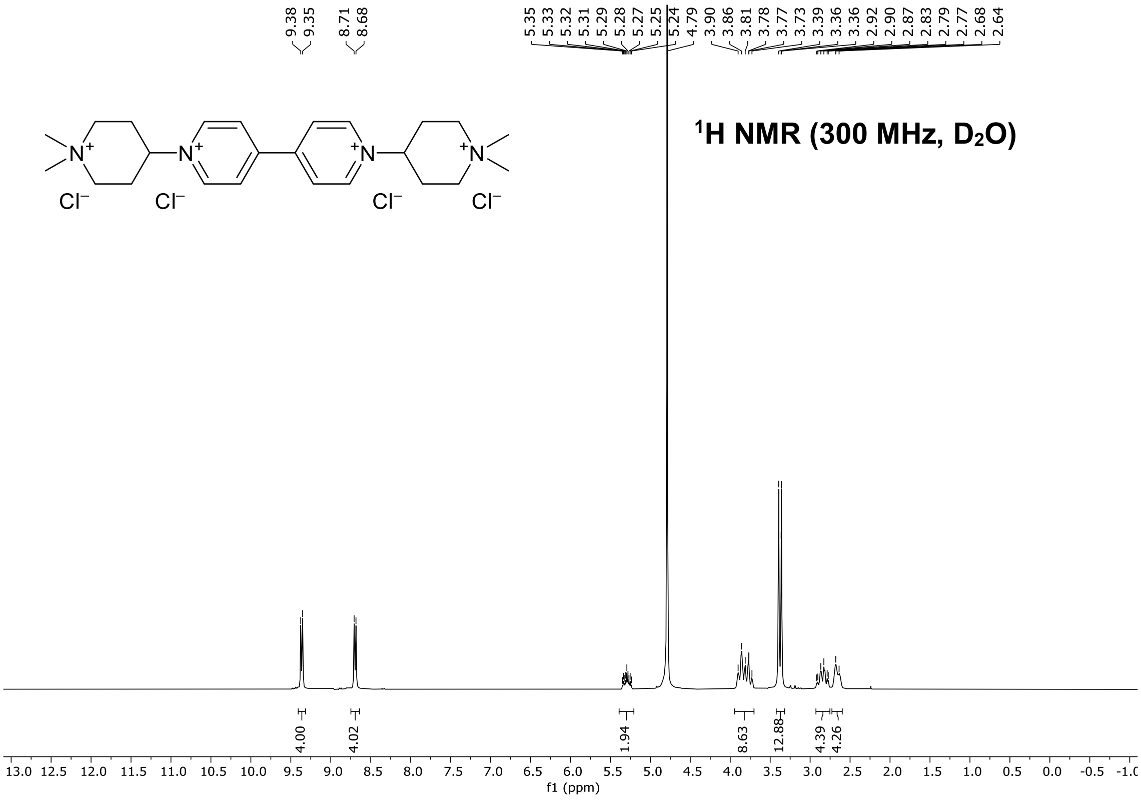

^{13}C-NMR spectrum.

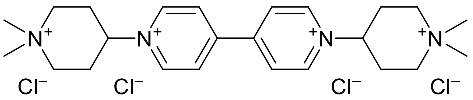

^{13}C NMR (75.4 MHz, D_2O)

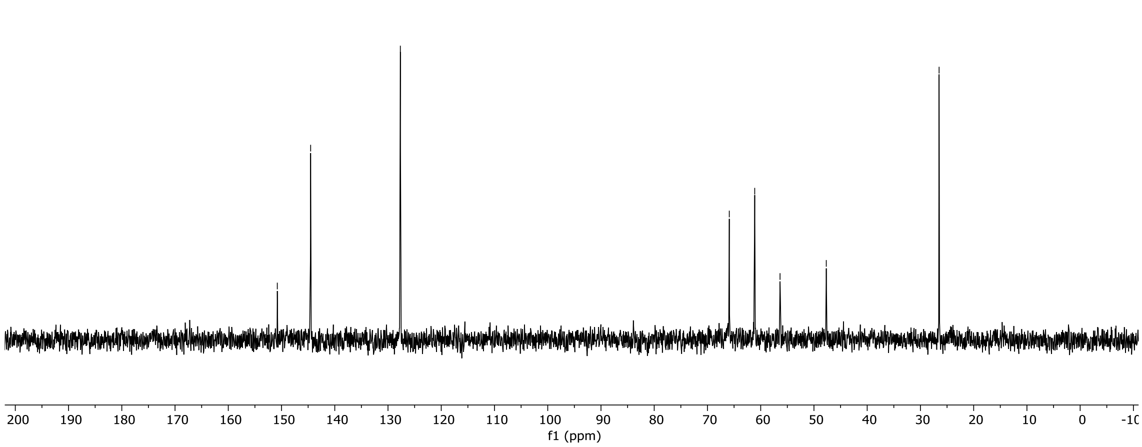

Section S2. Electrochemical Characterization

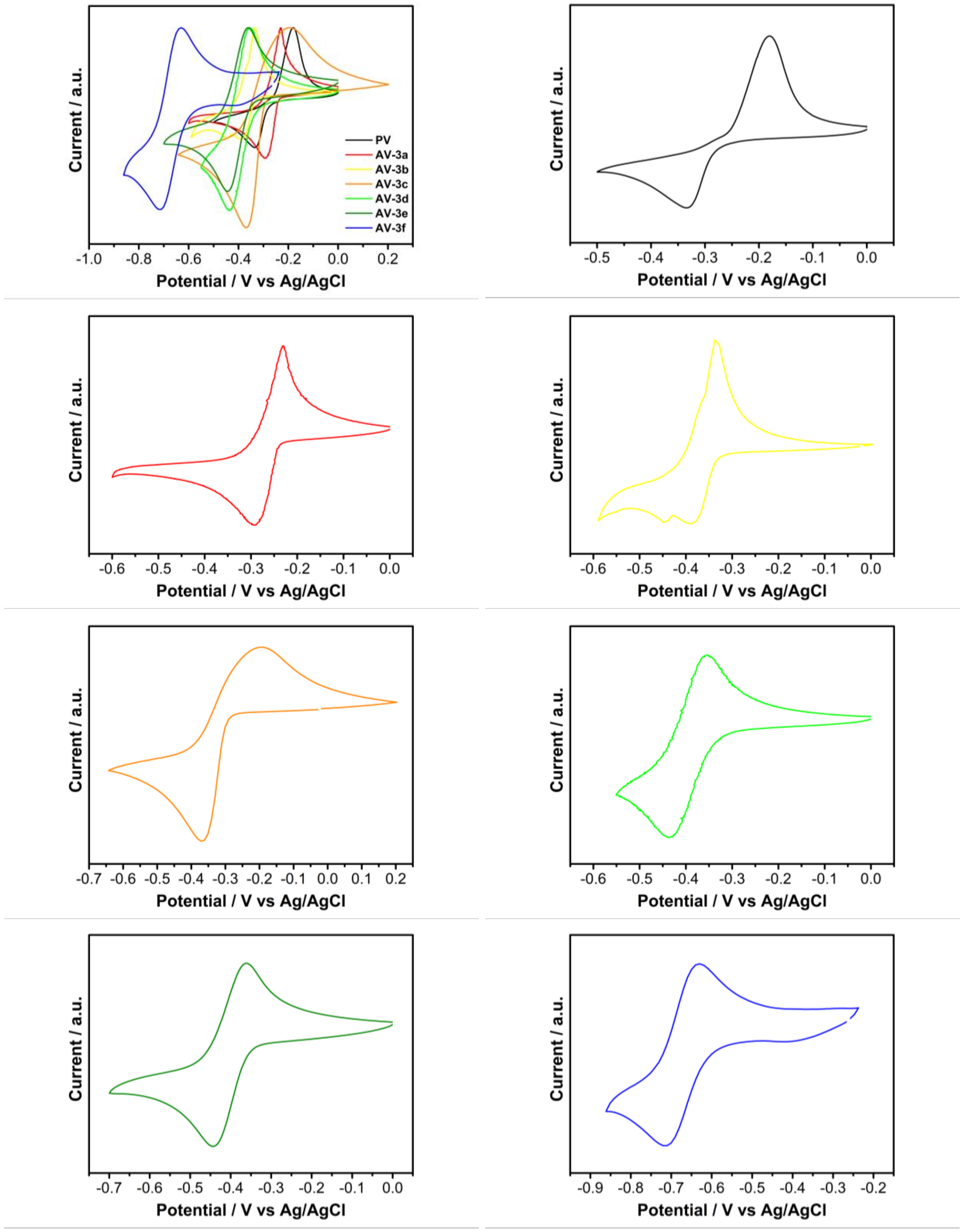

Figure S1. Cyclic voltammograms on glassy carbon and redox potentials of the aryl viologen derivatives (AVs) in 1 M KCl at pH 14. Scan rate: 100 mV/s. Concentration of AVs: 25 mM. The pH was adjusted by KOH.

The complete cyclic voltammetry curve (CV) of aryl viologen **B-2,5-DHPV** (**AV-3f**) in 1 M KCl at pH 14 (Figure S2) presented two reversible reduction process corresponding to the redox pairs of AV-3f^{2+}/AV-3f$^{\bullet+}$ (−0.68 V) and AV-3f$^{\bullet+}$/AV-3f^{0} (−1.02 V).

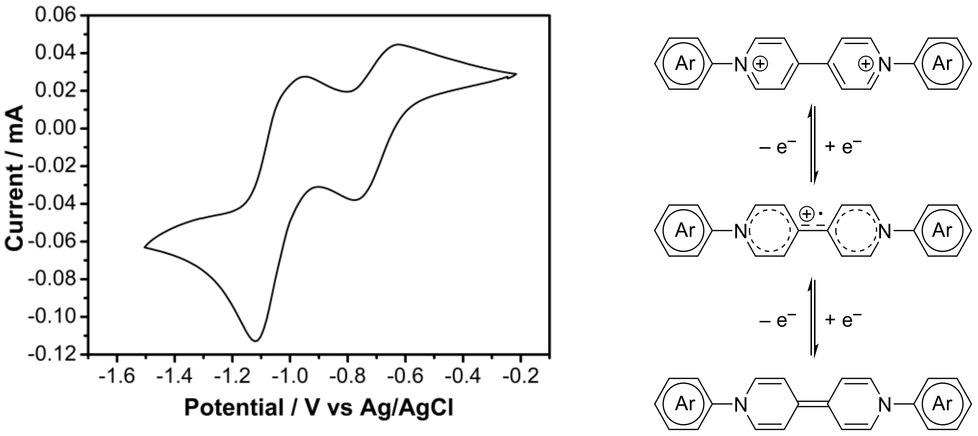

Figure S2. CV curve of 25 mM of **B-2,5-DHPV** (**AV-3f**) in 1 M KCl at pH 14. Scan rate: 100 mV/s. The pH was adjusted by KOH.

Section S3. Crossover Evaluation

We attribute the total capacity loss to chemical degradation and not to anolyte crossover. The contribution of the anolyte **B-2,5-DHPV** crossover to the capacity fading was negligible as we have estimated *via* UV-Vis spectrophotometric analysis of the receiving catholyte reservoir from a battery cell filled with 0.2 M of **B-2,5-DHPV** in 1 M KCl and 0.8 M KOH in the anolyte reservoir, and solely 1 M KCl and 0.8 M KOH in the receiving catholyte reservoir (Fig. S3).

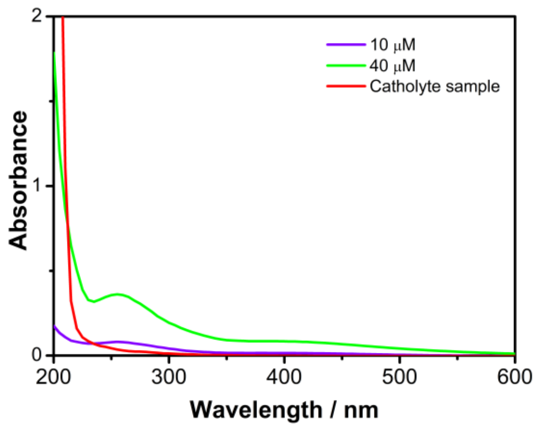

Figure S3. UV-Vis spectra of **B-2,5-DHPV** (**AV-3f**) at various concentrations and the receiving catholyte reservoir sample after 60 days.

For comparison, we evaluated the crossover of **(SPr)₂V** in equivalent experimental conditions (Fig. S4).

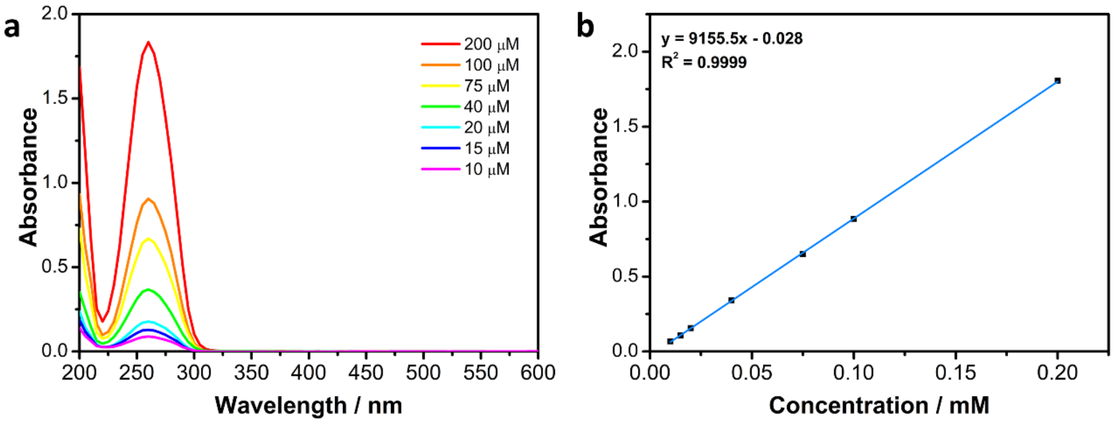

Figure S4. (a) UV-Vis spectra of **$(SPr)_2V$** at various concentrations and (b) the resulting calibration curve.

Permeability determination.

The permeabilities were determined by the following equation:[12]

$$P = \frac{\ln\left(1 - \frac{2C_c}{C_a}\right) \cdot \left(-\frac{V \cdot l}{2A}\right)}{t}$$

where C_c is the electroactive species molar concentration in the catholyte reservoir, C_a is the is the electroactive species molar concentration in the anolyte reservoir (0.2 M), V is the volume of the receiving catholyte reservoir (0.41 mL), l is the Nafion® membrane thickness (50.8 μm), and t is the elapsed time ($5.184 \cdot 10^6$ s).

The resulting measured permeability values determined from the data in Figure S3 and Figure S4 were $6.35 \cdot 10^{-11}$ $cm^2 \cdot s^{-1}$ at most for **B-2,5-DHPV**, and $1.07 \cdot 10^{-7}$ $cm^2 \cdot s^{-1}$ for **$(SPr)_2V$**.

Section S4. Estimation of Diffusion Coefficient

Figure S5 shows the results obtained for CV experiments performed at different scan rates for **B-2,5-DHPV** and the corresponding Randles−Sevcik plot.

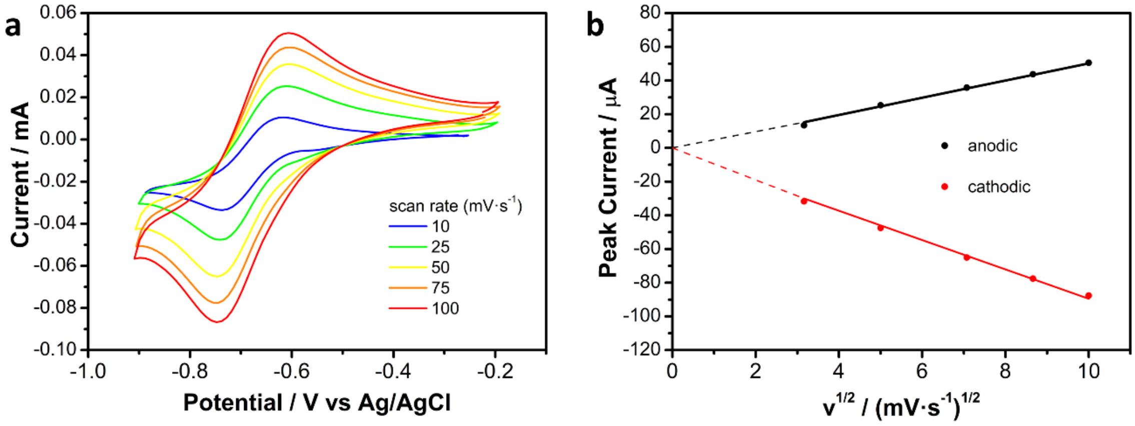

Figure S5. Electrochemical kinetics for aryl viologen **B-2,5-DHPV** (**AV-3f**): (a) CVs at different scan rates for the first reduction step and (b) the resulting Randles−Sevcik plot: peak current i_p as a function of the square root of the scan rate $v^{1/2}$ for the CV data set at 25 mM concentration of **B-2,5-DHPV** (**AV-3f**) in 1 M KCl at pH 14.

Diffusion coefficient determination.

The diffusion coefficient was determined by following the Randles−Sevcik equation:[13,14]

$$i_p = 0.446 \cdot n \cdot F \cdot A \cdot C^0 \cdot \left(\frac{n \cdot F \cdot v \cdot D_0}{R \cdot T}\right)^{1/2}$$

where i_p is the peak current (A), n is the number of electrons, A is the electrode surface area (cm^2), C^0 is the electroactive species concentration ($mol \cdot cm^{-3}$), D_0 is the diffusion coefficient ($cm^2 \cdot s^{-1}$), and v is the scan rate ($V \cdot s^{-1}$).

For experiments performed at 298.15 K (25 °C), the equation becomes (F = 96485.3321 $C \cdot mol^{-1}$; and R = 8.314462 $J \cdot mol^{-1} \cdot K^{-1}$):

$$i_p = 2.6847 \times 10^5 \cdot n^{3/2} \cdot A \cdot C^0 \cdot {D_0}^{1/2} \cdot v^{1/2}$$

A linear fit ($i_p = \mathrm{slope} \cdot v^{1/2}$) yields slope for the cathodic peaks of $-25.64 \cdot 10^{-5}$ $A \cdot V^{1/2}$ (R^2 = 0.9985), which gives a diffusion coefficient of $2.98 \cdot 10^{-7}$ $cm^2 \cdot s^{-1}$ for **B-2,5-DHPV**.

Section S5. Plot of the Capacity (mAh) versus time (days)

Figure S6 shows the performance of the full flow cell having 12 mL 0.2 M **B-2,5-DHPV** // 45 mL 0.3 M $K_4Fe(CN)_6$ in 1 M KCl and 0.8 M KOH (pH 14) using Ar overpressure in the negative compartment with 0.2 M of **B-2,5-DHPV** as anolyte in an alkaline AORFB.

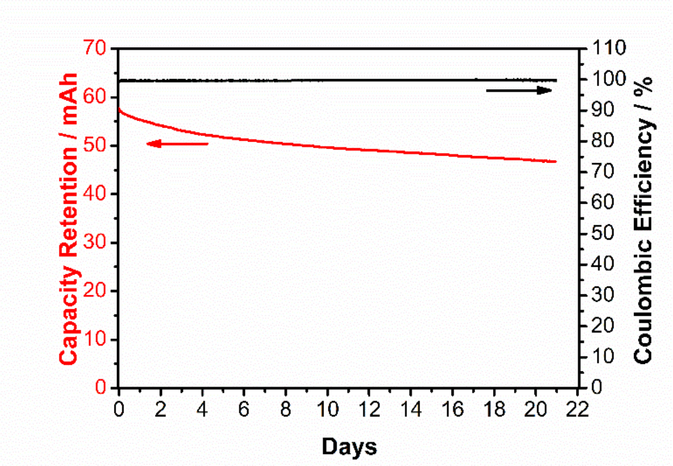

Figure S6. Performance of the full flow cell **B-2,5-DHPV** // $K_4Fe(CN)_6$ in 1 M KCl and 0.8 M KOH (pH 14). Evolution of the charge capacity of the flow cell upon cycling during 21 days. The cell was first charged/discharged at 30 mA·cm^{-2} until voltages reached 1.2 or 0.5 V and then was held at these voltages until the current density dropped to 1 mA·cm^{-2}.

References

1. Wang, S.; Li, S.; Xiong, J.; Lin, Z.; Wei, W.; Xu, Y. Near-Infrared Photothermal Conversion of Stable Radicals Photoinduced from a Viologen-Based Coordination Polymer. *Chem. Commun.* **2020**, *56* (54), 7399–7402. https://doi.org/10.1039/d0cc02193h.
2. Buyukcakir, O.; Je, S. H.; Talapaneni, S. N.; Kim, D.; Coskun, A. Charged Covalent Triazine Frameworks for CO_2 Capture and Conversion. *ACS Appl. Mater. Interfaces* **2017**, *9* (8), 7209–7216. https://doi.org/10.1021/acsami.6b16769.
3. Roztocki, K.; Formalik, F.; Bon, V.; Krawczuk, A.; Goszczycki, P.; Kuchta, B.; Kaskel, S.; Matoga, D. Tuning Adsorption-Induced Responsiveness of a Flexible Metal-Organic Framework JUK-8 by Linker Halogenation. *Chem. Mater.* **2022**, *34* (7), 3430–3439. https://doi.org/10.1021/acs.chemmater.2c00249.
4. Stasi, M.; Monferrer, A.; Babl, L.; Wunnava, S.; Dirscherl, C. F.; Braun, D.; Schwille, P.; Dietz, H.; Boekhoven, J. Regulating DNA-Hybridization Using a Chemically Fueled Reaction Cycle. *J. Am. Chem. Soc.* **2022**, *144* (48), 21939–21947. https://doi.org/10.1021/jacs.2c08463.
5. Van Antwerpen, P.; Prévost, M.; Zouaoui-Boudjeltia, K.; Babar, S.; Legssyer, I.; Moreau, P.; Moguilevsky, N.; Vanhaeverbeek, M.; Ducobu, J.; Nève, J.; Dufrasne, F. Conception of Myeloperoxidase Inhibitors Derived from Flufenamic Acid by Computational Docking and Structure Modification. *Bioorganic Med. Chem.* **2008**, *16* (4), 1702–1720. https://doi.org/10.1016/j.bmc.2007.11.025.
6. Zhao, J.; Qu, X.; Wang, J.; Yan, B. Photophysical Tuning of Viologen-Based Metal-Organic Framework Hybrids via Anion Exchange and Chemical Sensing on Persulfate ($S_2O_8^{2-}$). *Ind. Eng. Chem. Res.* **2019**, *58* (40), 18533–18539. https://doi.org/10.1021/acs.iecr.9b04049.
7. Liu, J. J.; Guan, Y. F.; Lin, M. J.; Huang, C. C.; Dai, W. X. Luminescent Coordination Polymer with Conjugated Lewis Acid Sites for the Detection of Organic Amines. *Cryst. Growth Des.* **2015**, *15* (10), 5040–5046. https://doi.org/10.1021/acs.cgd.5b01026.
8. Gong, T.; Yang, X.; Sui, Q.; Qi, Y.; Xi, F. G.; Gao, E. Q. Magnetic and Photochromic Properties of a Manganese(II) Metal-Zwitterionic Coordination Polymer. *Inorg. Chem.* **2016**, *55* (1), 96–103. https://doi.org/10.1021/acs.inorgchem.5b01888.
9. Wu, Y.; Xu, J.; Qin, X.; Xu, J.; Liu, X. Dynamic Upconversion Multicolour Editing Enabled by Molecule-Assisted Opto-Electrochemical Modulation. *Nat. Commun.* **2021**, *12* (1), 1–7. https://doi.org/10.1038/s41467-021-22387-7.
10. Biedermann, F.; Scherman, O. A. Model System for Studying Charge-Transfer Interactions. *J. Phys. Chem.* **2012**, *116* (9), 2842–2849. https://doi.org/10.1021/jp2110067.
11. Chen, N.; Jiang, Q.; Song, F.; Hu, X. Robust Piperidinium-Enriched Polystyrene Ionomers for Anion Exchange Membrane Fuel Cells and Water Electrolyzers. *ACS Energy Lett.* **2023**, *8* (10), 4043–4051. https://doi.org/10.1021/acsenergylett.3c01402.
12. Xie, W.; Cook, J.; Park, H. B.; Freeman, B. D.; Lee, C. H.; McGrath, J. E. Fundamental Salt and Water Transport Properties in Directly Copolymerized Disulfonated

Poly(Arylene Ether Sulfone) Random Copolymers. *Polymer* **2011**, *52* (9), 2032–2043. https://doi.org/10.1016/j.polymer.2011.02.006.
13. Bard, A. J.; Faulkner, L. R. Potential Sweep Methods *Electrochemical Methods: Fundamental and Applications*, 2nd ed, Wiley, **2001**; pp 226–260.
14. Elgrishi, N.; Rountree, K. J.; McCarthy, B. D.; Rountree, E. S.; Eisenhart, T. T.; Dempsey, J. L. A Practical Beginner's Guide to Cyclic Voltammetry. *J. Chem. Educ.* **2018**, *95* (2), 197–206. https://doi.org/10.1021/acs.jchemed.7b00361.
